# A Type VII-secreted toxin enables inter-mycobacterial competition

**DOI:** 10.1038/s41467-026-74429-7

**Published:** 2026-06-23

**Authors:** Samuel T. Benedict, Kieran Bowran, Eunice K. E. Lee, Jean-Lou Reyre, Huda Ahmad, Aaron Franklin, Nicole A. Mietrach, Cheng-Ruei Han, Jia Mun Chan, Abigail J. Layton, Emmanuele Severi, Eleanor R. Boardman, Kamilla Anochshenko, Gregory Goudge, Simon G. Caulton, Todd L. Lowary, Andrew L. Lovering, Manuel Banzhaf, Elisabeth C. Lowe, Tracy Palmer, Patrick J. Moynihan

**Affiliations:** 1 School of Biosciences, University of Birmingham, Birmingham, B15 2TT UK; 2 Newcastle University Biosciences Institute, Medical School, Newcastle University, Newcastle upon Tyne, NE2 4HH UK; 3 Institute of Biological Chemistry, Academia Sinica, Nangang, Taipei, Taiwan; 4 Northumbria University, School of Geography and Natural Sciences, Newcastle upon Tyne, NE1 8ST UK; 5 Institute of Biochemical Sciences, National Taiwan University, Taipei, Taiwan; 6 Department of Microbiology and Immunology, Schulich School of Medicine and Dentistry, The University of Western Ontario, London, Ontario, Canada

**Keywords:** Bacteriology, Glycobiology, Bacterial secretion, Bacterial structural biology

## Abstract

Most bacteria have evolved mechanisms to compete with other bacteria, often through the specialised secretion of proteinaceous toxins. However, mycobacteria have not previously been reported to engage in this form of competition. The thick and unusual mycobacterial cell wall, comprised of peptidoglycan, arabinogalactan and mycolic acids, is generally thought to be highly protective to these bacteria. Here, we show that some mycobacteria can use endo-D-arabinanases of the GH183 family for inter-bacterial competition. These microorganisms secrete an endo-D-arabinanase effector via the type VII secretion system (T7SS) that cleaves the arabinogalactan layer of the mycobacterial cell envelope. We describe the molecular basis for this activity using structural biology and biochemistry, and identify a protein family that protects the bacterium from the activity of this toxin. The widespread presence of genes potentially encoding similar T7SS-secreted toxins in the *Mycobacteriales* suggests extensive inter-mycobacterial competition.

## Abstract (web summary)

Mycobacteria have not yet been reported to engage in interbacterial competition via secretion of proteinaceous toxins. Here, Benedict et al. show that some mycobacteria can use their type-VII secretion system for this purpose, by secreting enzymes that cleave the arabinogalactan layer of the mycobacterial cell envelope.

## Introduction

Mycobacterial species are often considered in isolation, likely due to the paradigm of *Mycobacterium tuberculosis*, which is typically thought of as existing in monoculture during infection. However, most mycobacteria are considered non- or opportunistically-pathogenic and instead live in complex ecosystems^1,2^. In these environments, it can be expected that they will need to compete for resources. This could include specialised mechanisms for metal or nutrient acquisition or through direct interference competition.

In other bacteria, this evolutionary pressure has driven the emergence of several mechanisms to deliver diverse anti-bacterial proteinaceous effectors. In Gram-negative organisms, competition is often driven by the type VI secretion system, where toxic effectors are secreted into nearby cells through a contractile apparatus resembling a phage baseplate^3–5^. In Bacillota, proteinaceous effectors are targeted to bacterial cells via the Type VIIb secretion system, which lacks a similar contractile apparatus^6–9^.

Up to five Type VIIa secretion systems (T7SSa) are encoded by mycobacteria, termed ESX-1-5. ESX-1 is an essential virulence factor in *M. tuberculosis*, and its inactivation is the key attenuating mutation in the *M. bovis* BCG vaccine strain^10^. The largest group of T7SSa substrates is the PE/PPE proteins, which are named for conserved motifs in their N-terminus and comprise 10% of the *M. tuberculosis* proteome^11^. These proteins heterodimerise through their N-termini to form helical bundles^12^. A conserved WxG motif in the PPE protein and a proximal YxxxD/E in the PE partner likely form a composite signal sequence^13,14^. PE/PPE proteins are associated with all ESX loci except ESX-4. Conversely, *M. tuberculosis* CpnT, the precursor of tuberculosis necrotising toxin, a host-targeting NAD^+^-glycohydrolase, requires ESX-4 for secretion^15,16^. The CpnT N-terminus is predicted to adopt a PE/PPE-like fold despite sharing little detectable sequence similarity.

The Mycobacteriales cell wall is unusual amongst bacteria in that it is a tripartite structure consisting of peptidoglycan, arabinogalactan, and mycolic acids^17^. This structure is essential under most conditions and, combined with other cell envelope components, poses a substantial permeability barrier. We recently discovered the glycoside hydrolase family 183 (GH183), which likely enables arabinogalactan remodelling through cleavage of the d-arabinan backbone^18^. In this study, we demonstrate that some mycobacteria have weaponised a sub-family of these enzymes, which is T7-secretion dependent and mediates inter-mycobacterial competition.

## Results

### Mycobacteria encode a predicted type VII secreted GH183

**Fig. 1:**
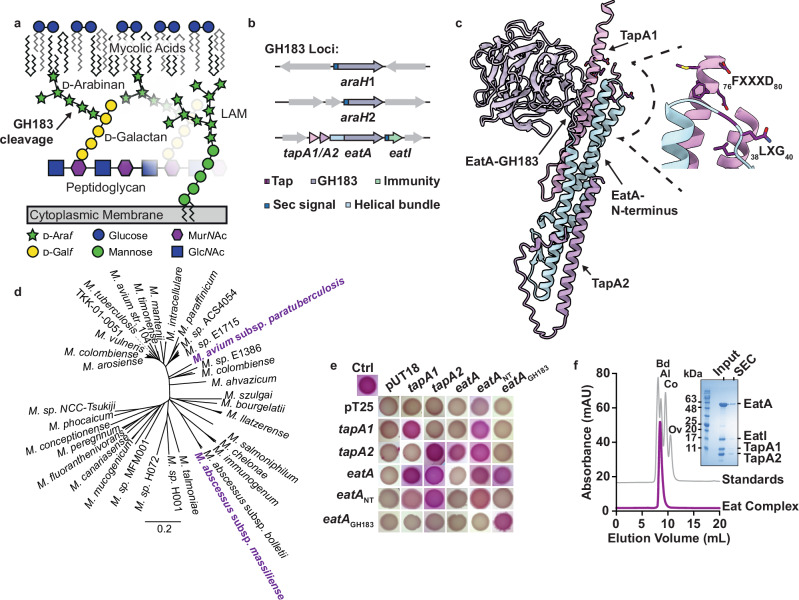
Mycobacteria encode a GH183 enzyme in a toxin-like locus. **a** Simplified view of the mycobacterial cell wall, highlighting the site of GH183 activity. **b**The *M. abscessus* subsp. *massiliense* str. GO 06 genome encodes two conserved housekeeping GH183 enzymes (MYCMA_0617, MYCMA_0180) and a third copy (MYCMA_14050) with a locus structure including two small globular proteins (MYCMA_14055,60) encoded 5’ to the GH183 gene and a previously unassigned ORF downstream. The housekeeping GH183 enzymes (AraH1, AraH2) and the unannotated ORF each have a predicted N-terminal Sec-secretion signal; the unannotated ORF also has a predicted lipo-box. **c** The AlphaFold3 prediction for TapA1 and TapA2 in complex with EatA reveals a predicted complex similar to other Type VII-secreted substrates. AlphaFold 3 prediction statistics are presented in Supplementary Fig. 2. **d** An unrooted FoldTree using Foldseek score of cluster A0A7X5ZCJ1 demonstrates that the *eat* locus is distributed across the *Mycobacterium* genus in both slow-, and fast-growing organisms. Enzymes characterised here are shown in purple. **e** Bacterial-two-hybrid results for *tap* genes and *eatA* illustrating interactions between the EatA N-terminus and both Tap proteins. Each gene was encoded as a fusion on either the pT25 or pUT18 plasmid and co-transformed. NarG-T18 and T25-NarJ were used as a positive control (top left)^75^. The resulting strains will produce red pigmentation on MacConkey agar when bait and prey proteins interact in this system. **f** Analytical size-exclusion of co-expressed genes from the *eat* locus demonstrating they elute as a single, high molecular weight complex. The input and eluted fractions were analysed by SDS-PAGE (inset), which showed the presence of all locus members in a single complex. Expected molecular weights: EatA − 56.9 kDa, EatI − 16.1 kDa, TapA1 − 12.7 kDa, TapA2 − 8.6 kDa, Complex − 94.3 kDa, blue dextran (Bd) − 200 kDa, aldolase (Al) − 158 kDa, conalbumin (Co) − 75 kDa, ovalbumin (Ov) − 44 kDa. Data are representative of two biological replicates.

Arabinogalactan forms a covalent bridge between the peptidoglycan and mycolic acid outer membrane and is essential for the viability of mycobacteria (Fig. 1a)^19–21^. This polysaccharide is phylogenetically restricted to the *Mycobacteriales*, and d-arabinan is only known to be essential in this group of bacteria. The recently discovered GH183 endo-d-arabinofuranases specifically target the α−1,5 linkages within d-arabinan, and two of these housekeeping enzymes are found in virtually all mycobacteria^18,22^. During the work leading to the identification of these enzymes, we recognised that some mycobacteria, including some *Mycobacterium abscessus* strains, encoded an additional GH183 homolog (Fig. 1b).

In *M. abscessus* subsp. *massiliense* str. GO 06 this protein, MYCMA_14050, is encoded at a locus with two small globular proteins (MYCMA_14055/60) and an unannotated open reading frame coding for a small lipoprotein (Fig. 1b). This locus organisation is reminiscent of the T7SSb toxin locus structure in Bacillota^6^. In Bacillota, T7SSb-secreted toxins usually have an N-terminal LxG domain, and they are co-encoded with helical partner proteins, termed **L**xG **a**ccessory **p**roteins (Lap) required for toxin secretion, and an immunity protein to prevent self-intoxication^7^. Given this locus organisation and the phylogenetic restriction of d-arabinan, we speculated that MYCMA_14050 may be involved in inter-mycobacterial competition. For reasons discussed later, we have renamed MYCMA_14050 as **E**sx-**a**ssociated **t**oxin A (EatA), with MYCMA_14055/60 designated TapA1/A2 (**T**oxin **a**ccessory **p**rotein), and the unannotated ORF as EatI (**Eat I**nhibitor).

AlphaFold 3 prediction of EatA revealed an elongated α-helical N-terminal domain predicted to interact with TapA1 and TapA2 (Fig. 1c)^23^. Comparison of this prediction with structures of a T7SSa PE/PPE complex or toxin/accessory protein complexes from T7SSb is consistent with the EatA complex including appropriately positioned T7SS-targeting motifs (Fig. 1c, Supplementary Fig. 1, 2)^14,24^. Phylogenetic analysis of the EatA N-terminal region revealed it to cluster with helical domains from Bacillota T7SSb substrates rather than T7SSa substrates (Supplementary Fig. 1b). The presence of a GH183 domain and low sequence identity in the N-terminal helical domain of EatA complicated BLAST identification of orthologs. To circumvent this, we analysed the Foldseek cluster A0A7X5ZCJ1, to which EatA belongs, using FoldTree to determine the distribution of EatA (Fig. 1d)^25,26^. To our surprise, EatA orthologs are found only in mycobacterial species, including one *M. tuberculosis* isolate, and approximately 60 species possess an EatA-like protein. This included both fast- and slow-growing environmental and pathogenic mycobacteria and several uncultured isolates (Fig. 1d).

### TapA1 and TapA2 form a complex with EatA_Mab_

T7 secreted toxins from the Bacillota usually form complexes with small helical partners, which are then secreted^24,27^. This prompted us to test the interaction of all cognate pairs of EatA, EatA_GH183_ (amino acid (AA) 185–526), EatA_N-term_ (AA 2–180), and TapA1/TapA2 in a bacterial-two-hybrid assay (Fig. 1e)^28^. This revealed TapA1 and TapA2 to interact with both full-length EatA and its N-terminal domain, but not with each other, consistent with the AlphaFold3 prediction (Fig. 1c)^23^. The 2-hybrid system also suggested some components, EatA_GH183_, EatA_N-term_, TapA1, and TapA2, but not EatA could self-associate. To further test this, we expressed the entire locus in *E. coli,* isolating complexes via a His_6_ tag on EatA_Mab_ and a twin Strep-tag on TapA1. This resulted in a high-molecular-weight complex as determined by size exclusion chromatography, which contained all the proteins encoded at the locus, the elution volume (9 mL) is consistent with a monomer of this complex when compared to standards, however full structural characterisation would be required to confirm this (Fig. 1f).

### The Eat locus encodes an inhibitory protein

**Fig. 2:**
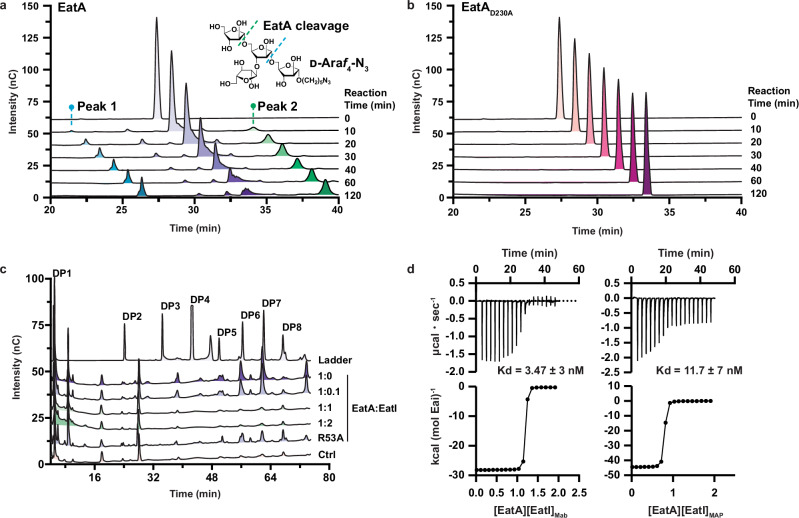
EatA proteins are GH183 family members and are inhibited by EatI. EatA_Mab_ (**a**) or EatA_MabD228A_ (**b**) (1 μM) were incubated with 50 µM d-Ara*f*_4_-N_3_ (structure inset) and at the indicated timepoints reactions were analysed by ion chromatography with pulsed amperometric detection. Representative chromatograms of three biological replicates have been offset in the x-axis to aid visualisation. Ion-chromatography mass spectrometry analysis of peak 1 and 2 are presented in Supplementary Fig. 4. **c** EatA_Mab_ (1 μM) was incubated either on its own or with the indicated molar ratios of EatI_Mab_ and 1 mg·mL^−1^ arabinogalactan from *M. smegmatis* mc^2^155. For EatI_MabR53A_, a 1:1 molar ratio was used. Representative chromatograms of three biological replicates are presented. The ladder is comprised of l-arabinofuranose oligosaccharides with the indicated degree of polymerisation (DP) and the signal has been scaled by half to aid visualisation. **d** Isotherms of binding of EatA proteins to their cognate EatI proteins. Data is representative of two biological replicates. The top panel shows raw titration thermal changes in response to ligand addition, whilst the bottom panels show integrated peaks. Calculated Kd is shown between each panel.

Glycoside hydrolases from the same families can catalyse very different activities, and so to confirm the catalytic function of EatA, we expressed and purified the GH183 domain from *M. abscessus* (AA 183-526). We then conducted hydrolysis assays using arabinogalactan and a synthetic d-arabinan oligosaccharide (d-Ara*f*_4_-N_3_; Fig. 2; Supplementary Fig. 3a, b). This confirmed the identity of EatA as an endo-d-arabinofuranase. Using the synthetic substrate, the enzyme had a specific activity of 0.027 ± 0.003 U·mg^−1^ (Supplementary Fig. 3b) and we observed no product formation when the catalytic acid/base was replaced with alanine (D228A) (Fig. 2b). Upon mass-spectrometry analysis of the reaction products generated using this synthetic substrate, we confirmed ɑ−1,5 d-arabinofuranase activity for EatA (Supplementary Fig. 4). The enzyme also displayed activity against arabinogalactan purified from *Mycobacterium smegmatis* mc^2^155 (Fig. 2c).

EatI is predicted to be a lipoprotein with no previously assigned function. We purified the *M. abscessus* protein (AA 25–172) without its predicted signal sequence and found that at an approximately 1:1 molar ratio of EatA_Mab_:EatI_Mab_, EatA activity was completely inhibited (Fig. 2c). Given its predicted localisation to the cell envelope, we speculated that EatI binds directly to EatA, and so we combined the proteins and analysed them by size-exclusion chromatography (Supplementary Fig. 5). This revealed formation of a higher molecular weight complex, which we determined had a Kd of 3.47 ± 3 nM and a 1:1 molar ratio by isothermal titration calorimetry (Fig. 2d).

Given the conservation of this locus in many *Mycobacteriales* members, we purified the EatA GH183 domain (MAP44135_3721; EatA_MAP_; AA 214–540) and EatI (MAP44135_3722; EaI_MAP_; AA 22–170) from *Mycobacterium avium* subsp. *paratuberculosis* strain DSM 44135, which is a slow-growing mycobacterial species and the causative agent of Johne’s disease in ruminants. We confirmed similar biochemical functions for these proteins (Fig. 2d, Supplementary Fig. 3c), with EatI_MAP_ inhibiting EatA_MAP_ with a Kd of 11.7 ± 7 nM (Fig. 2d). We also found that the predicted immunity proteins were not inhibitory and did not form complexes for non-cognate pairs of *M. abscessus* and *M. avium* subsp. *paratuberculosis* proteins (Supplementary Fig. 3d, 5).

### Molecular basis of Eat inhibition

**Fig. 3:**
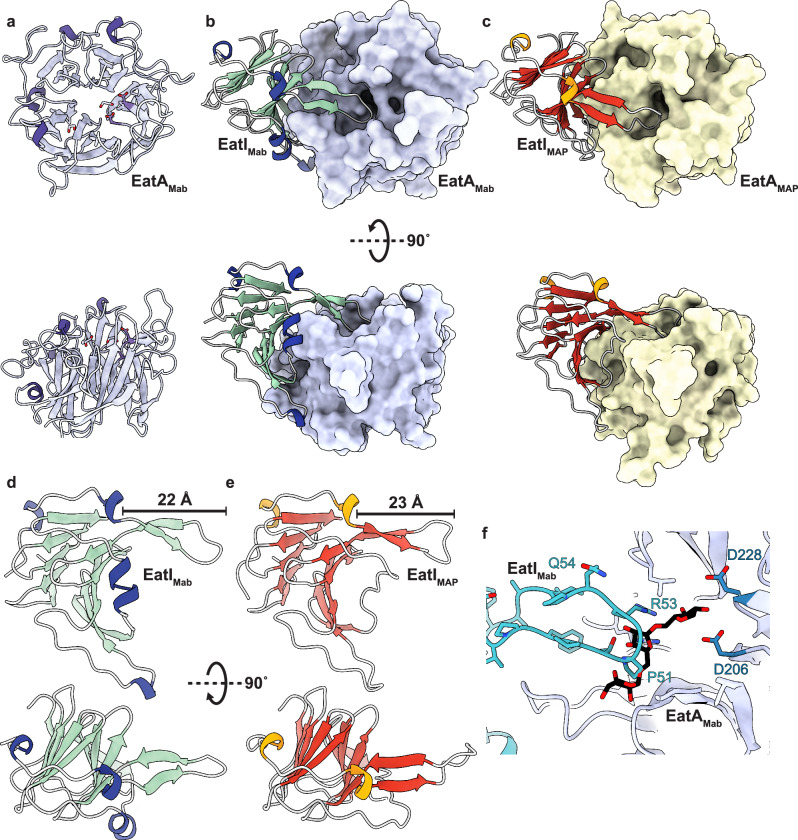
Molecular basis for EatI inhibition of EatA toxins. **a** A 2.06 Å X-ray crystal structure of apo-EatA_Mab_ reveals a five-bladed β-propeller fold. **b** The 1.7 Å structure of EatA-EatI_Mab_ reveals a 1:1 stoichiometry with the placement of an inhibitory loop from EatI_Mab_ in the active site of EatA_Mab,_ which is shown in the same pose as in panel (**a**) in lilac with surface representation. **c** The 1.65 Å EatA-EatI_MAP_ complex is presented in the same orientation as the EatA-EatI_Mab_ structure, showing a similar overall fold and pose for both proteins. Structural alterations at the interface between these preclude cross-inhibition by non-cognate immunity proteins (Supplementary Fig. 3d, 7). The overall fold of EatI from *M. abscessus* subsp. *massiliense* (**d**, green) and *M. avium* subsp. *paratuberculosis* (**e**, orange) is a β-sandwich with an elongated hairpin that forms the protein’s inhibitory loop. **f** When aligned with the substrate (substrate shown in black) complex of EndoMA1 (PDB: 8IC1), it becomes evident that the inhibitory loop of EatI would preclude productive binding of the substrate. ɑ-Helices and β-strands are presented in contrasting colours to aid in visualisation.

To understand the molecular basis of EatA activity, we solved a 2.06 Å apo structure of the GH183 domain of EatA_Mab_ (A.A. 181-524; Fig. 3a; Supplementary Table 1). There were two monomers in the asymmetric unit, making a dimer via non-crystallographic symmetry which is unlikely to be physiologically relevant. Overall, each monomer resembles a five-bladed β-propeller. The two monomers are bound to a total of 11 ions that have been modelled as potassium, which are derived from the crystallisation conditions. Similarly, the proteins are bound to several ethylene glycol molecules, with two bound in each active site (Fig. 3a, Supplementary Fig. 6a). The protein is structurally similar to the GH domain of EndoMA1 (PDB: 8IC1; Supplementary Fig. 6b), a characterised GH183 from *Microbacterium arabinogalactanolyticum* with an overall RMSD of 5.48 Å^22,29^. There are equivalent substrate-binding residues in EatA to most of those coordinating a modified tetra-d-arabinofuranoside ligand in EndoMA1 (Supplementary Fig. 6c). However, the loop containing P141 in EndoMA1 is significantly truncated in EatA, leading to a more open active site (Supplementary Fig. 6d–f). This alignment supports the designation of D228 as the catalytic acid/base and D206 as the nucleophile in EatA.

We next solved a 1.7 Å structure of the GH183 domain of EatA_Mab_ in complex with EatI_Mab_ (Fig. 3b; Supplementary Table 1). Two complexes were observed in the asymmetric unit, which are nearly identical in structure (RMSD of 0.36 Å). The overall complex supports our size exclusion and isothermal titration calorimetry data, demonstrating a 1:1 stoichiometry for the GH183 and immunity domains, resulting in a buried surface area of 1628.5 Å ^2^^30^. The immunity protein is comprised of a 10-strand β-sandwich made up of two β-meanders (Fig. 3b, d). To our knowledge, this is the first experimentally determined structure for this fold. Compared to the EatA_Mab_ apo structure, the binding of EatI_Mab_ induces a few conformational changes (Supplementary Fig. 7a–d). Most notably, the small helical turn in EatA_Mab_ formed by P439 − N441 becomes unwound (Supplementary Fig. 7b), shifting the Cɣ of P439 by 7.4 Å towards EatI_Mab_ to make hydrophobic interactions with V46, Y60 and Y125 (Supplementary Fig. 7c). This positions EatA_Mab_ D438 to form a salt bridge with H124 of EatI_Mab_ (Supplementary Fig. 7d). A second salt bridge is formed between R445 of EatA_Mab_ and E115 of EatI_Mab_. This rearrangement allows an extended β-hairpin of EatI_Mab_ to project approximately 22 Å into the active site of EatA_Mab_ placing the conserved R53 in the active site (Fig. 3b, d, f; Supplementary Fig. 7g). When compared to the EndoMA1 substrate complex (PDB: 8IC1; Supplementary Fig. 6b), it becomes clear that the extended hairpin of EatI_Mab_ blocks the predicted −1 to −3 substrate-binding subsites, precluding catalytic activity (Fig. 3f). While structurally unrelated, this inhibitory mechanism is reminiscent of BliPI and II inhibition of TEM-1 and several families of lysozyme inhibitors^31–34^.

To help understand why EatI proteins were not cross-protective in vitro, we also solved a 1.65 Å structure of the *M. avium* subsp. *paratuberculosis* EatA_MAP_–EatI_MAP_ complex (Fig. 3c, e). The structure is broadly similar to the EatA_Mab_–EatI_Mab_ complex (overall RMSD of 2.58 Å) with a buried interface of 1465.2 Å^2^. The binding of the EatI_MAP_ to EatA_MAP_ has several distinct features, however, likely explaining the lack of EatI cross-inhibition. EatA_MAP_ lacks the small interface helix (EatA_Mab_ AA 439-441), which is unwound in the EatA_Mab_–EatI_Mab_ complex. Instead, this loop is truncated by four residues to six amino acids in EatA_MAP_. Similarly, in EatI_Mab_, residues 119 − 124 form a helix absent in EatI_MAP_ (Fig. 3d, e, Supplementary Fig. 7e, f). This shifts the equivalent loop in EatI_MAP_ (AA 113 − 122) by 3.8 Å, which would clash with the unwound interface helix in EatA_Mab_. The net result of these structural changes is that the inhibitory β-hairpin in EatI_MAP_ is shifted by approximately 4 Å as it passes over the side of EatA_MAP_ relative to equivalent residues in the EatA–EatI_Mab_ complex (Supplementary Fig. 7g). Surprisingly, despite this shift, the arginine residue (R53_Mab_/52_MAP_) at the tip of the loop is placed in a nearly identical location in both structures indicating that the mechanism of substrate occlusion is likely the same for both complexes (Supplementary Fig. 7g). Supporting this, substituting R53 with alanine reduces EatI_Mab_ inhibition of EatA_Mab_ (Fig. 2c).

### EatI and EatA are secreted proteins

**Fig. 4:**
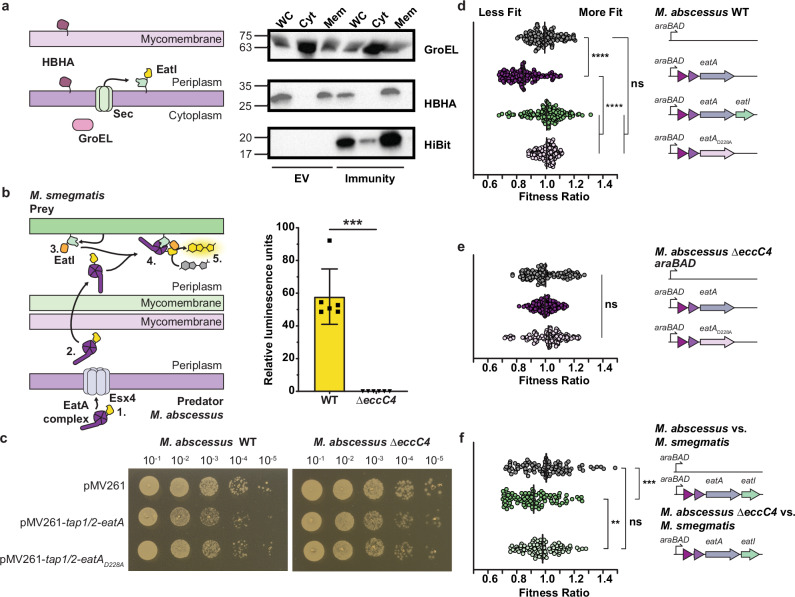
EatA proteins have toxic effects in mycobacteria. **a** Western blot of fractionated cells expressing EatI fused to the HiBit/pep86 component of split nanoluciferase. GroEL corresponds to samples blotted with an anti-GroEL antibody, HBHA is heparin binding hemagglutinin antigen, and HiBit detected using an antibody raised against this component of split-nanoluciferase. Data are representative of three biological replicates. Uncropped blots are presented in Supplementary Fig. 14. **b** The sequence encoding the LgBit/11 s component of split nanoluciferase was fused to *eatI* and expressed from a plasmid in *M. smegmatis* and the HiBit/pep86 component was similarly fused to *eatA*_*D228A*_ and expressed in *M. abscessus* wild type or ∆*eccC4*. Either wild-type or ∆*eccC4 M. abscessus* was combined with the *eatI-*expressing *M smegmatis* and luciferase activity was detected by adding NanoGlo substrate and measurement in a plate reader. Luciferase activity was quantified by raw relative light units measured in a plate reader following addition of NanoGlo substrate. Significance determined using two-tailed Welch’s t-test, *p* = 0.0004; *n* = 3 biological replicates. **c** *M. abscessus* ATCC 19977 or *M. abscessus* ATCC 19977 ∆*eccC4* were transformed with a pMV261 plasmid containing the indicated *eatA* locus components under control of the strong *groEL* constitutive promoter, grown in broth and then serially diluted onto tryptic soy agar. A small bubble is present in the 10^−5^ dilution of pMV261-*tapA1/2-eatA*. **d** *M. abscessus* ATCC 19977 or a (**e**) ∆*eccC4* derivative were transformed with a pMYBAD plasmid containing the indicated components of the *eatA* locus under the control of an arabinose inducible promoter. These were pinned (*n* = 128 biological replicates; error bars represent standard deviation; p values are <0.0001 for all significant comparisons) onto tryptic soy agar with either 1% d-glucose or l-arabinose, grown for 48 h and imaged. Fitness is measured as a ratio of the colony opacity grown in non-inducing (d-glucose) or inducing (l-arabinose) conditions. **f** The *M. abscessus* WT or ∆*eccC4* strain containing either an empty vector or the complete toxin locus was mixed at a 4:1 ratio with *M. smegmatis* mc^2^155 pTEC27-tdTomato. These were pinned (*n* = 96 biological replicates, error bars represent standard deviation; p values: ** = 0.0047; *** = 0.0001) onto solid media, and colony fluorescence was used as a proxy for *M. smegmatis* growth. Fitness is measured as the ratio of normalised colony fluorescence in inducing (l-arabinose) over non-inducing (d-glucose) conditions. The experimental workflow, colony images and control fluorescence data are presented in Supplementary Figs. 10–12. Significance for panels **d**–**f** was determined using a one-way analysis of variance with Tukey’s multiple comparisons test to compare differences from the control condition.

The EatI protein harbours a predicted Sec signal peptide with an appropriately placed cysteine residue for lipidation. To confirm this, we expressed *eatI* with a fusion to the pep86 portion of the split nanoluciferase and analysed whole cell, cytoplasmic and membrane fractions by western blot. The protein was heavily enriched in the membrane fraction, consistent with its expected localisation (Fig. 4a).

Based on sequence analysis it is expected that EatA will be secreted via the Type VII secretion system. ESX-4 is the ancestral T7SS system in mycobacteria, and its expression is induced by cell-cell contact^35,36^. We, therefore, hypothesised that this system was most likely driving secretion of EatA. We inactivated *M. abscessus* ATCC 19977 ESX-4 by deleting *eccC4*, which encodes part of the core T7SS apparatus^37–40^. To confirm that ESX-4 secretion was eliminated, we assessed secretion of EsxU in both wild-type and the ∆*eccC4* strain (Supplementary Fig. 8). We next generated a plasmid encoding *tapA1, tapA2* and *eatA*_D228A_-pep86 which we transformed into *M. abscessus* ATCC 19977. We then transformed an *eatI-*11s expressing plasmid into *M. smegmatis* mc^2^155. As outlined in Fig. 4b, a functional nanoluciferase enzyme is produced through interaction of the pep86 and 11s nanoluciferase components, driven by EatA and EatI binding, in the periplasm of *M. smegmatis*. To test this, we mixed the strains and allowed them to settle in liquid media to encourage cell-cell contact. We then measured nanoluciferase activity, which was produced when the cells were combined and was ESX-4 dependent (Fig. 4b). These data demonstrate that EatI and EatA are secreted and that EatA requires ESX-4 for delivery into target cells.

### EatA is toxic to mycobacteria via an ESX-4-dependent mechanism

*M. abscessus* ATCC 19977 lacks the *eatA* locus, and so to generate an experimentally tractable system, we constructed a plasmid containing the *M. abscessus* subsp. *massiliense* str. GO 06 *eat* locus lacking the *eatI* gene under the control of a constitutive, strong promoter^41^. We transformed this plasmid into the ATCC 19977 strain where it drove a 10-fold reduction in growth, as compared to either a control empty vector strain or a D228A catalytic mutant (Fig. 4c). Despite repeated attempts, we were unable to produce the immunity protein using strong promoters because of toxicity (Supplementary Fig. 9). This has been observed for immunity lipoproteins of the T7SSb system previously, and is likely a consequence of out-titrating secretion of housekeeping proteins via the Sec system^7^.

We next sought to develop a method where we could quantify either self, or inter-bacterial competition based on this system. To obtain a lower-level of expression and avoid toxicity, we turned to the pMYBAD-K plasmid backbone^42^. This system relies on a tuneable l-arabinose inducible promoter, where we generated a series of *eat* locus constructs containing just the toxin and *tap* genes or the entire locus. Recognising that antagonism by the T7SS is contact-dependent in the Bacillota, and mycobacteria are non-motile and heterogeneous we wanted to develop a method to quantify competition on solid agar^7^. Inspired by approaches used for chemical genomics, we developed a high-throughput pinning assay to overcome these limitations by quantifying mycobacterial competition and testing for EatA-induced self-killing (Fig. 4d-f; workflow in Supplementary Fig. 10a)^43,44^.

We pinned strains onto agar containing either repressor (d-glucose) or inducer (l-arabinose) and imaged the resulting colonies under standardised conditions. For self-intoxication, fitness is quantified as the ratio of opacity (Fig. 4d; Supplementary Fig. 11a) or size (Supplementary Fig. 10b,c) of colonies derived from the same source culture, pinned onto inducer divided by repressor plates. In this way we can directly measure the impact of induction of the *eat* locus in large numbers of biological replicates at a time. In wild-type *M. abscessus* ATCC 19977 we observed a significant decrease in fitness in an *eatA-*dependent fashion (Fig. 4d). This decrease required a catalytically competent EatA and was rescued by including EatI (Fig. 4d). These data allow us to conclude that expression of EatA is toxic to *M. abscessus* and can be counteracted by expression of the cognate immunity protein.

Having established earlier that EatA secretion is ESX-4 dependent (Fig. 4b), we repeated our assays in the ∆*eccC4* background. Self-killing was eliminated in our spot-dilution assay in this genetic background (Fig. 4c), suggesting that this system is required for *eatA*-mediated growth inhibition. Furthermore, using the colony pinning approach, we observed no *eatA*-dependent toxicity in the ∆*eccC4* strain, further supporting our designation of secretion of this protein as ESX-4 dependent (Fig. 4e; Supplementary Fig. 11b).

We next sought to determine if EatA could drive inter-species competition, so we made a prey strain by generating a strain of *M. smegmatis* mc^2^155 harbouring the pTEC27 plasmid, which encodes the gene for tdTomato under the control of a constitutive promoter^45^. To confirm that tdTomato fluorescence correlated with the growth of *M. smegmatis*, we pinned the prey strain at a range of starting OD_600nm_ values, demonstrating correlation between fluorescence and the quantity of live bacteria (Supplementary Fig. 12). We then combined this fluorescent prey strain with the *M. abscessus* strain harbouring the complete *eat* locus at a 4:1 ratio and, as above, pinned them onto solid agar. To measure the fitness of the prey strain, we calculated the total fluorescence per colony when grown on l-arabinose or d-glucose and expressed this as a fitness ratio (Fig. 4f). Similar to the self-toxicity analysis above, we observed an EatA-dependent reduction in fitness for the prey strain (Fig. 4f). From this we conclude that EatA can drive inter-mycobacterial competition. These data demonstrate that mycobacteria produce a T7SS-dependent toxin that enables inter-mycobacterial competition.

### Putative T7SS-dependent interbacterial toxins are broadly distributed in the Actinomycetota

**Fig. 5:**
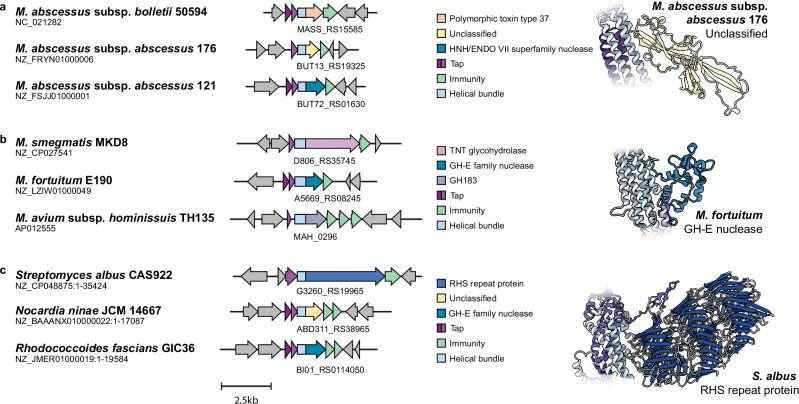
Actinomycetota bacteria encode diverse T7-secreted toxins. Representative *eat* loci from **a** *M. abscessus* **b** *Mycobacterium* and **c** Actinomycetota strains. The helical bundle and upstream *tap*-like gene(s) are conserved features of these loci, which encode a diverse range of predicted toxin payloads (Supplementary Table 3). Genes are colour-coded for predicted function. The number of *tap*-like genes and predicted immunity proteins varies by locus. On the right are representative AlphaFold 3 structural predictions for a toxin domain from each grouping.

Having established that mycobacteria utilise T7SS secretion for interbacterial competition, we sought to determine the diversity of putative toxic effectors in the Actinomycetota. Given the large number of available genomes and the varied environments in which it grows, we first analysed 946 *M. abscessus* strains available on NCBI. To determine the distribution of loci across *M. abscessus* and whether they followed the gene synteny described above, we queried our dataset for Tap-like proteins, yielding 1439 unique protein sequences (summarised in Supplementary Table 3). Many of these were confirmed to be encoded in similar operons to the *eat* locus in discrete locations within the genome (Fig. 5a).

These putative toxins span many predicted functions, including many known toxin families (Fig. 5a, Supplementary Table 3). The most commonly identified families were α/β hydrolase, nuclease HNH/ENDO superfamilies, NlpC/P60 family and TNT domain family (Supplementary Table 3). Proteins with no predicted function constituted 47% of all protein sequences. These proteins typically have between 400 and 1000 residues, accompanied by one or two Lap/Tap-like encoding genes that mirror what was previously described for T7SSb substrates. Downstream-encoded immunity proteins tended to possess structural features reflecting the subcellular compartment of the corresponding proposed toxin target. For example, predicted extracellular targeting toxins would co-occur with adjacent immunities with predicted lipoprotein signal sequences or transmembrane helices.

Expanding this analysis to the *Mycobacteriales* highlights the presence of diverse Esx-dependent toxins within *Mycobacterium* and across the phylum (Fig. 5b, c), suggesting that T7SS-mediated inter-(myco)bacterial competition is a conserved feature.

## Discussion

In this study, we identify that mycobacteria use a T7-secreted proteinaceous effector to drive inter-mycobacterial competition. The EatA family targets cell wall arabinogalactan of the *Mycobacteriales*, which we have shown reduces fitness in a manner that requires catalytically active enzyme. Our identification of EatI proteins also shows how these bacteria protect themselves from self-killing, using a newly characterised protein fold as a specific and tight-binding EatA inhibitor. This killing mechanism is conceptually similar to the use of peptidoglycan-targeting effectors in other systems, such as Type VI-mediated interbacterial competition^46^.

While our data broadly support the presence of Type VII secreted toxic effectors, the extent of killing observed is reduced relative to that reported for comparable systems. Several factors may account for this discrepancy. The technical challenges associated with working with mycobacteria, which are highly hydrophobic and nonmotile, complicate the study of a putatively contact-dependent mechanism. These characteristics limit opportunities for productive cell to cell interactions under standard culturing conditions and preclude the use of many assays that are routinely applied in more technically tractable systems. In addition, the regulatory complexity of the ESX-4 system remains poorly understood, and it is therefore likely that our predator strains produce insufficient levels of the secretion machinery or associated components to enable efficient toxin translocation. Finally, the number and diversity of effectors encoded across mycobacterial genomes suggest that individual effectors may not be sufficient for full toxicity. This is conceptually similar to the requirement for both a peptidoglycan targeting LysA and a mycolic acid targeting LysB for efficient prey lysis by mycobacteriophages^47,48^. Finally, a less likely possibility is that EatA mediates toxicity by enabling secretion or up-take of other toxins and proteins secreted by the Type VII machinery, rather than through its activity directly.

The EatA system appears to be unusual amongst anti-bacterial effector proteins because it is biochemically restricted to a single phylum. Arabinogalactan is only found in the *Mycobacteriales* and is essential for their viability under most growth conditions. Indeed, its biosynthesis is the target of the front-line anti-tubercular drug ethambutol^49–53^. The existence of the *eatA* locus encouraged us to look for similar loci across mycobacteria, revealing numerous and diverse predicted T7-secreted toxic effectors. Excitingly, many of these loci encode predicted effector domains with no known function, pointing towards potential new targets for anti-mycobacterial therapy.

The competition for resources provides an enormous opportunity for evolution to shape bacterial genomes. The presence of ubiquitous T7-secreted toxin-mediated interference competition in the *Mycobacteriales* reframes our thinking of the evolutionary pressures placed on this phylum. *M. tuberculosis*, for example, is frequently thought of as living in isolation, and yet all strains of *M. tuberculosis* encode potential anti-bacterial T7-secreted toxins (Supplementary Fig. 13). Indeed, this points to a possible evolutionary origin for tuberculosis-necrotising toxin, which is also ESX-4-dependent^16^.

Our study raises several important unresolved questions. For EatA to be toxic, the protein must access the periplasm of target cells. Many of the putative toxins we have identified are predicted to have cytoplasmic targets and correspondingly have predicted cytoplasmic immunity proteins. The mechanism by which these toxins transit the predator and prey cell envelopes remains unclear. A model by which the small Esx proteins facilitate tuberculosis-necrotising toxin transport has been proposed; however, it is unclear how this would facilitate transport across three biological membranes and so would need to be validated in an inter-bacterial competition context^54^. It also remains to be determined if T7-secreted toxic effectors remain bound to their N-terminal carrier domain or if this is cleaved during secretion. Additionally, the mechanism by which toxin-secretion is regulated remains unclear and understanding this will be key to improving killing efficiency. Finally, the range of prey organisms that the T7 secretion system can target remains unknown but will significantly impact microbial ecology.

## Methods

### Strains and growth conditions

Unless stated otherwise, all chemicals and reagents were purchased from Sigma-Aldrich. *Escherichia coli* T7 SHuffle or BL21-DE3 (New England Biolabs) were used to express recombinant proteins, and *E. coli* 10-β was used for plasmid maintenance. *E. coli* strain M15 was used for over-production of the EatA-EatI-Tap protein complex. *M. smegmatis* mc^2^155 and *M. abscessus* ATCC 19977 were used in predation and self-toxicity experiments transformed through electroporation with the appropriate plasmids. *E. coli* strains, up to 100 mL, were grown in lysogeny broth. *E. coli* cultures used for protein purification were grown in terrific broth at 37 °C in 2 L baffled flasks at 180 RPM. Routine cultures of *M. smegmatis* mc^2^155 and *M. abscessus* ATCC 19977 were grown in tryptic soy broth (TSB) media for liquid cultivation and tryptic soy agar (TSA) for solid growth at 37 °C. Luciferase growth assays were conducted in Sauton’s medium with 1% glycerol^55^. For mycobacteria, apramycin, kanamycin and hygromycin selection were at 50 μg/mL.

### AlphaFold and FoldTree analysis

For protein structure prediction, amino acid sequences were inputted into the AlphaFold 3 server and run using default settings^23^. Structural data were analysed using ChimeraX^29^. To identify EatA orthologs, the UniProt ID for EatA_Mab_ (A0A0U0ZL93) was input into the AlphaFold Clusters server, which identified Cluster A0A7X5ZCJ1^25^. This was then input into the Google Colab notebook for FoldTree, and the resulting tree was produced using FoldSeek score^26^. The resulting data were imported into Geneious Prime 2024.0.7 and are presented as an unrooted tree.

### Bacterial-2-Hybrid assay

pT25 or pUT18 plasmid backbones encoding fusions to the appropriate bait or prey genes were transformed into *E. coli* BTH101^28^. These strains were spotted onto MacConkey agar supplemented with 1% d-maltose, 25 μg/mL chloramphenicol and 125 μg/mL ampicillin and grown at 30 °C until colonies were visible and control colonies were visibly pink before being photographed. Formation of a complex between the T18 and T25 components of adenylate cyclase leads to lactose fermentation by the parental strain and pink pigmentation on MacConkey agar^28^.

### Cloning

The expression plasmids for EatA_Mab_ (AA 183–526), EatI_Mab_ (AA 25–172), EatA_MAP_ (AA214–540), and EatI_MAP_ (AA 22–170) were synthesised and codon-optimised for *E. coli* expression by Twist Biosciences in a pET28a vector, including a hexahistidine tag and GSG linker on the N-terminus of EatA_Mab_, EatI_Mab_, and EatI_MAP_, proteins, and at the C-terminus of EatA_MAP_. Inserts for the pMV261 plasmids were synthesised by Genewiz and then subcloned into pMV261 using BamHI and XhoI restriction sites. All primers are listed in Supplementary Table 2.

### Purification of the Eat complex

A plasmid was constructed by ligating the *tapA1*/*A2*-*eatA*-*eatI* locus from *M. abscessus* subsp. *massiliense* str. GO 06, including a hexahistidine and twin-strep affinity tags to enable co-expression of the Eat complex, which we termed pqe70-*eatI*-*tapA1*-*tapA2*-TS-*eatA*-His_6_. This plasmid was transformed into *E. coli* M15 [pREP4] was grown in 2 L of terrific broth to OD_600nm_ 0.6-1.0. Expression was induced using 1 mM IPTG for 4 h at 37 °C before harvesting and lysis in 50 mM HEPES pH 7.5, 150 mM NaCl, and 25 mM imidazole. Following lysis, we used a 3-step purification. This included a first purification using Ni-NTA resin in which the protein was bound and washed in the same buffer before batch elution with an increase to 500 mM imidazole. Fractions positive for the complex components were dialysed against 100 mM Tris-HCl pH 8, 150 mM NaCl, 1 mM EDTA and loaded onto a Strep-Trap XT column. The protein was eluted in BXT buffer (IBA), concentrated, and dialysed into 25 mM HEPES pH 7.5, 150 mM NaCl before size exclusion chromatography with a HiLoad 26/600 200 pg column or for analytical runs, a Superdex 75 increase column in the same buffer.

### Purification of MAB and MAP EatA/EatI

*E. coli* T7 SHuffle (New England Biolabs) were transformed with pET28a vector by heat shock and plated on lysogeny broth agar (VWR) plates supplemented with 50 μg/mL kanamycin. One plate of transformants was scraped into one litre of terrific broth before growing at 37 °C to an OD_600nm_ of 0.6-1.0 before cooling in cold water and inducing with 250 μM IPTG overnight at 16 °C. Cells were centrifuged at 4500 x *g* for 20 min before resuspending in a buffer containing 25 mM HEPES pH 8, 400 mM KCl, and 10 mM imidazole pH 8 with cOmplete protease inhibitor tablets (Roche). This cell suspension was lysed by sonication before clarifying the soluble fraction by centrifugation at 40,000 x *g* for 40 min at 4 °C. Crude proteins were isolated by immobilised metal affinity chromatography using cOmplete nickel resin (Roche). Fractions were assessed for purity by SDS-PAGE and then dialysed against 3 L of 25 mM HEPES pH 8, 200 mM KCl before further purification by size exclusion chromatography using an Äkta Pure system using a HiLoad 26/600 200 pg column. Following size exclusion, proteins were concentrated using spin concentrators before snap freezing in liquid N_2_ and stored at −80 °C before use. Protein concentrations were determined using the Pierce BCA protein assay kit (ThermoFisher Scientific) per the manufacturer’s instructions using the microplate protocol.

### Crystallography and data collection

For complex structures, toxin and immunity were mixed and equilibrated on ice for 20 min, ensuring a molar excess of immunity protein. Samples were then concentrated and further purified by size exclusion chromatography using an Äkta Pure system using a HiLoad 26/600 200 pg column (Cytiva) to remove excess immunity protein. The resulting purified complex was then concentrated. The crystallisation was conducted using the sitting drop vapour diffusion method at with a final volume of 2 μL. Sealed plates were incubated at 18 °C until crystal formation was observed.

For MAP_complex_, crystals formed in JCSG+ condition A10 (0.2 M ammonium nitrate pH 6.3; 20% PEG 3350) (Molecular Dimensions) in a 1:1 protein: precipitant ratio after approximately one week. Crystals were indexed in space group P2_1_2_1_2_1_ and were irradiated on beamline IO3 at Diamond Light Source at 100 K. For MAB_complex_, crystals formed in Memgold2 condition C1 (0.075 M Magnesium chloride hexahydrate; 0.1 M Sodium cacodylate pH 6.5; 30 % w/v PEG 2000 MME) (Molecular Dimensions) in a 2:1 protein: precipitant ratio after approximately one week. Crystals were indexed in the space group P2_1_. For EatA_Mab_ apo, crystals formed in JCSG+ condition G10 (0.15 M potassium bromide; 30% PEG 3000-MME) (Molecular Dimensions) in a 1:1 protein: precipitant ratio after approximately one month. Crystals were indexed in the space group C222_1_. All crystals were irradiated on beamline IO3 at Diamond Light Source at 100 K.

All steps of phasing, solving, and structural refinement of datasets were carried out in the PHENIX suite (version 1.21rc1-5127-000). Phases were solved using PHASER using the appropriate AlphaFold predicted structure and pruned to high confidence regions using the ‘Process Predicted Model’ package as a search model. Following phasing, successive rounds of manual and automated refinement using COOT (version 0.9.8.8) and PHENIX.refine, respectively, were carried out to build final models.

### Isothermal titration calorimetry

Prior to analysis, aliquots of protein were dialysed for 3 h against 25 mM HEPES pH 8 with 200 mM KCl, to ensure identical buffers. EatA proteins were added to the chamber at 20 μM, and EatI was added to the syringe at 200 μM. Analyses were performed on a MicroCal PEAQ-ITC calorimeter at 25 °C using nineteen 2 μL injections, with a reference power of 10 µcal. Data were analysed using MicroCal PEAQ-ITC Analysis Software (version 1.41).

### Analytical size exclusion chromatography

Proteins were normalised to a concentration of 150 μM in 25 mM HEPES pH 8 and 200 mM NaCl. Chromatography was carried out at a flow rate of 0.8 mL•min^−1^ on an Äkta pure system fitted with a Superdex 75 increase column (Cytiva), which had previously been equilibrated into the same buffer as above. The Eat and EatI proteins were mixed in a 1:1 molar ratio with either their cognate or non-cognate inhibitor proteins or buffer for single protein runs. Fractions were collected using the inline fraction collector and then subjected to 15% SDS-PAGE to analyse composition.

### Kinetic analysis of EatA

To assess the degradation kinetics of EatA and EatA_D228A_ on d-Ara*f*_4_-N_3_, enzymes (1 µM) were incubated with 50 µM d-Ara*f*_4_-N_3_ at 37 °C in 50 mM citrate-phosphate buffer (pH 7). Reactions were conducted in technical triplicates and were terminated by adding an equal volume of 100 mM sodium hydroxide. The reaction products were analysed by IC-PAD-MS following the methods described below. A standard curve was generated to determine d-Ara*f*_4_-N_3_ concentrations, and data visualisation was performed using GraphPad Prism 10.4.1.

### Ion chromatography with pulsed amperometric detection and mass spectrometry (IC-PAD-MS)

The tetra-arabinofuranoside ligand (d-Ara*f*_4_-N_3_) used in kinetics investigations was synthesised using methods described previously^56,57^. The degradation kinetics of d-Ara*f*_4_-N_3_ and oligosaccharides derived from enzymatic arabinogalactan digestion were analysed using Ion Chromatography with Pulsed Amperometric Detection and mass spectrometry (IC-PAD-MS). The analyses were performed on an ICS-6000 system equipped with a CarboPac PA-300 anion exchange column (ThermoFisher). Detection was facilitated by a gold working electrode and a PdH reference electrode, using a standard Carbo Quad waveform. Buffer A consisted of 100 mM NaOH, while Buffer B was composed of 100 mM NaOH and 0.5 M sodium acetate. For arabinogalactan product analysis, each sample was injected and run at a constant flow rate of 0.25 mL·min^−1^ for 100 min using the following method: 0–10 min, isocratic elution with 100% Buffer A; 10–70 min, a linear gradient to 60% Buffer B; and 70–80 min, 100% Buffer B. The column was subsequently washed for 10 min with 500 mM NaOH, followed by re-equilibration for 70 min in 100% Buffer A.

For d-Ara*f*_4_-N_3_ analysis, the following method was applied: 0–5 min, isocratic elution with 100% Buffer A; 5–40 min, a linear gradient to 60% Buffer B; and 40–50 min, 100% Buffer B. The column was washed for 5 min with 500 mM NaOH, followed by re-equilibration in 100% Buffer A for 10 min. Commercially available l-arabinofuranoside-oligosaccharides (degree of polymerisation = 1–8) were used as standards (Megazyme) at a concentration of 25 µM.

Mass spectrometry was conducted in negative ion mode using electrospray ionisation using the following parameters: 3400 V, ion transfer tube temperature of 325 °C, scan range of 300-2500 *m/*z. Data analyses were performed using ThermoFisher Freestyle 1.8 SP2 software, and final graphs were created using GraphPad Prism 10.4.1.

### EatA inhibition assays

Inhibition kinetics were assessed by incubating EatA (1 µM) and its cognate immunity protein at varying concentrations (0.1 µM, 1 µM, 2 µM) with arabinogalactan (1 mg·mL^−1^) for 1 h at 37 °C in 50 mM citrate-phosphate buffer (pH 7). Prior to the reaction, EatA and the immunity protein were pre-incubated for 30 min at 4 °C in 50 mM citrate-phosphate buffer (pH 7). Reactions were conducted in technical triplicates and were terminated by adding an equal volume of 100 mM sodium hydroxide.

### Thin layer chromatographic enzyme assay

EatA and EatI proteins were normalised to 200 µM in 25 mM HEPES pH 8; 200 mM NaCl. EatA proteins were added to reactions at a final concentration of 5 µM. 1 mg•mL^−1^ arabinogalactan purified from *M. smegmatis* mc^2^155 was used as the substrate, and 50 mM NaCl and 20 mM Tris-HCl (pH 8) were included in the reactions. Reactions were incubated for 1 h at 37 °C before boiling for 10 min to deactivate the enzyme. Ten μL of the reaction mixture was then spotted on silica gel TLC plates (Merck) and developed twice in 2:1:1 butan-1-ol: acetic acid: water before spraying with orcinol (5 g orcinol in 375 mL ethanol, 107 mL water, 16.2 mL concentrated sulphuric acid) and charred to reveal products.

### Construction of Δ*eccC*_*4*_ mutants in *M. abscessus* ATCC 19977

*M. abscessus* mutants were generated using allelic exchange using the plasmid pKH-aac(3)-IV-dsRed2-katG^58^. Allelic exchange was achieved following the methods outlined in Rominski et al 2017. Briefly, the knockout vector was generated using HiFi assembly using primers to amplify 1.5 kb regions flanking the gene of interest. Competent *M. abscessus* cells were electroporated with 500 ng of knockout plasmid using a single pulse of 2.5 kV, 25 µF, 1000 Ω. Cells were recovered in TSB at 115 rpm at 37 °C for 4 h and plated onto LB agar containing 50 μg/mL apramycin. Plates were incubated at 37 °C until the formation of pink colonies, followed by screening for genetic crossover events. Candidates were streaked onto LB agar containing 32 μg/mL isoniazid and incubated at 37 °C until the formation of white colonies. Candidates were patched onto LB agar supplemented with either apramycin or isoniazid to confirm resistance phenotypes and screened by PCR and whole genome sequencing to confirm the loss of the target gene.

### Fitness assay

Fresh transformants of *M. abscessus* WT and Δ*eccC*_*4*_ were grown in TSB + 0.05% Tween-80 and 50 μg/mL kanamycin, pelleted by centrifugation (5000 x *g*; 10 min; room temperature) and resuspended in fresh TSB before diluting to OD_600nm_ = 0.1 and then diluting 1:10 serially five times. 5 μL spots of each dilution were dispensed onto TSA plates containing 50 μg/mL kanamycin and incubated for 72  h before imaging.

### Pinning assays

For pinning competition assays, the prey strain was *M. smegmatis* mc^2^155 transformed with pTEC27, an L5-integrating plasmid driving constitutive expression of tdTomato^45^. Predator strains were *M. abscessus* ATCC 19977 transformed with pMYBADC-K with no insert or the *eat* genomic locus, including the two *tap* genes, *eatA* and *eatI* protein under the control of an arabinose inducible promoter or derivatives thereof. Mycobacterial cultures of predator and prey strains were grown in TSB with 0.05% Tween-80 with selection as appropriate to late stationary phase (OD_600nm_ = 2-4). Cultures were then pelleted at 3000 x *g* for 10 min before resuspending in the original volume of fresh TSB with 0.05% Tween-80. The culture was then diluted to an OD_600nm_ of 0.7 for *M. abscessus* self-intoxication assays and 0.5 for competition pinning assays. 100 µL per well of resulting cell suspension was transferred to a 96-well source plate (Sterilin) and pinned in a 384-spot format using the Biomatrix BM3-BC robot in single-well plates (VWR) onto either TSB or 7H9 plates solidified with 2% agar including 1% l-arabinose or d-glucose and incubated at 37 °C for 48 h. Following incubation, the plates were imaged with an 18-megapixel Canon EOS Rebel T3i camera within the BM3-BC robot under controlled lighting. The S&P Imager (S&P Robotics, version 2.0.1.0) and EOS Utility (Canon, version 2.10.0.0) software programs were used for image processing.

### Colony Fluorescence Data

Fluorescent data were collected using a BMG Polarstar plate reader. The excitation and emission wavelengths were 544 nm and 590 nm, respectively. Analysis was completed using the following R packages: imager, magick, ggplot2, reshape2, and ggbeeswarm^59–61^. The R script used for fluorescence data analysis is available as Supplementary Software 1 and at https://github.com/Hudaahmadd/Fluorescence-Analysis. Fitness is calculated from total raw fluorescence per colony rather than per unit colony area. This means that the modest co-variation in colony size arising from prey killing is captured directly by the measurement and does not independently confound interpretation.

### Design and purification of anti-EccC_4_ antibody

Protein sequences of EccC_3_ (WP_005110740.1) and EccC_4_ (WP_005055990.1) were extracted from *M. abscessus* ATCC 19977 and aligned against EssC (Q932J9; residues 964 – 1479; *essC*_*1*_ variant strain^62^ from *Staphylococcus aureus* Mu50) to identify the analogous cytoplasmic DIII domain in the mycobacterial proteins as previously described^63^. All steps of gene synthesis, cloning, protein overexpression, and antibody production were carried out by GenScript. Briefly, mycobacterial genes were codon optimised and synthesised with the addition of an N-terminal hexahistidine-TEV fusion into pET28a(+), expressed within *E. coli* BL21 Star (DE3). Expression was induced with 500 μM IPTG for 16 h at 15 °C, 200 rpm. Purified protein was obtained from the cell lysate supernatant by nickel affinity chromatography per manufacturer’s guidelines, with protein confirmed using monoclonal mouse-α-His antibody (GenScript; Cat.No. A00186). Antibodies were raised in rabbits with three immunisation rounds, followed by ammonium sulphate precipitation.

### Purification and generation of α-EsxU antibodies

EsxU from *M. abscessus* was codon optimised and synthesised with the addition of an N-terminal hexahistidine-TEV fusion into pET28a(+) (GenScript) and subsequently transformed into *E. coli* BL21 (DE3). Cultures were grown until an OD_600nm_ of ~0.6 – 0.8. Cells were induced with 0.5 mM IPTG for 4 h at 37 °C, with 200 rpm orbital shaking. Protein overproduction was confirmed via SDS-PAGE. Cells were harvested by centrifugation at 4000 x *g*, with supernatants removed and pellets washed with PBS followed by resuspension in Buffer A supplemented with cOmplete™ mini EDTA-free protease inhibitor cocktail (Sigma-Aldrich). Cells were lysed by sonication and pelleted by centrifugation, with the resultant supernatant passed through a 0.45 µm filter. The filtered supernatant was loaded onto an equilibrated HisTrap FF 5 mL nickel Sepharose column (Cytiva), subsequently subjected to a denaturing wash with 50 mM Tris-HCl pH 7.5, 100 mM NaCl, 25 mM imidazole, 10% glycerol, 8 M urea, washed in the same buffer lacking urea and eluted with the same buffer using a gradient of imidazole to 500 mM. Peak fractions were pooled, visualised by SDS-PAGE and concentrated using a 3 kDa spin concentrator (ThermoFisher Scientific). The concentrated samples were dialysed in 50 mM Tris-HCl pH 7.5, 100 mM NaCl and 10% glycerol overnight at 4 °C. Dialysed samples were incubated with 1 mg of TEV protease and left rolling overnight at 4 °C. TEV protease-cleaved proteins were isolated through reapplication and reverse affinity nickel chromatography. Proteins were further purified by size exclusion chromatography with a Superdex 75 10/300 GL (Cytiva). Peak fractions were collected, concentrated and confirmed by SDS-PAGE.

Samples of EsxU were sent to Davids Biotechnologie to generate polyclonal antibodies. New Zealand rabbits were subjected to five protein immunisations over 63 days, followed by antigen-specific affinity purification. Antibodies were tested against purified protein samples to confirm activity and binding specificity.

### EsxU secretion assay

Cultures were grown in 250 mL flasks at 37 °C with shaking at 115 rpm in TSB supplemented with 100 μg/mL ampicillin to OD_600nm_ = 1. Following culture, cell pellets were collected, washed 3 times in PBS and resuspended at 30 OD units/mL before lysis by three rounds of bead beating using 0.1 mm glass beads, cooling samples on ice between rounds of agitation. Samples were then prepared in 1x Laemmli buffer (final concentration) before analysis. For supernatant fractions, culture supernatants were sterilised by passage through a 0.2 μm syringe filter. Supernatants were then precipitated with 10% (final concentration) trichloroacetic acid at 4 °C overnight. Pellets were then collected by centrifugation at 7000 x *g* before washing with ice-cold acetone. Remaining acetone was evaporated under airflow and pellets resuspended at 0.6 OD_600nm_ units/μL in 1x Laemmli buffer^64^. Samples were boiled and subjected to SDS-PAGE using precast 4-20% polyacrylamide gels (Bio-Rad). Gels were transferred to nitrocellulose membranes using wet transfer before washing, blocking for 1 h in 5% skim milk in TBST and incubation overnight in primary antisera (rabbit anti-EccC_4_; rabbit anti-EsxU; mouse anti-GroEL (BEI resources)) in 1% skim milk in TBST. Membranes were washed and then incubated in secondary antibody (Bio-Rad; goat anti-rabbit IgG 1706515; goat anti-mouse IgG 1706516; 1:5000 in Tris buffered saline) bands were visualised using chemiluminescence using Clarity™ ECL substrate (Bio-Rad).

### Dual nanoluciferase assay

All plasmids used in this assay were produced using HiFi isothermal assembly using Q5 DNA polymerase and HiFi mastermix (New England Biolabs). Starter cultures of strains were grown in TSB supplemented with 0.05% (w/v) tween-80 and 50 μg/mL kanamycin to stationary phase. Cultures were then diluted to OD_600nm_ = 0.25 and grown for 18 h in the same media. Cultures were then pelleted at 4000 x *g* and resuspended in PBS. Cell suspensions were then normalised to OD_600nm_ = 1 in PBS. Prey strains (*M. smegmatis* mc^2^155:: pMV261; *M. smegmatis* mc^2^155:: pMV261-EatI; *M. smegmatis* mc^2^155:: pMV261-EatI-11s) were mixed in a 4:1 ratio with predator strains (*M. abscessus* ATCC 19977:: pMV261-Eat-D228A-pep86; *M. abscessus* ATCC 19977 Δ*eccC4*:: pMV261-Eat-D228A-pep86). These mixed cultures were then diluted to OD_600nm_ = 0.5 and used to inoculate 100 μL final volume of Sauton’s media supplemented with 1% glycerol and kanamycin at an OD_600nm_ of 0.05 in white polystyrene flat bottom plates (Greiner). Plates were incubated at 30 °C for 26 h and then total luciferase signal quantified by addition of NanoGlo substrate (Promega). Total luminescence was then quantified using a FluoStar Omega plate reader (BMG LabTech) following incubation at 37 °C.

### EatI localisation assay

Localisation of the putative immunity protein, EatI, was confirmed by fractionating western blot. Briefly, 50 mL of strains were grown to an OD_600nm_ of 7 before pelleting. Whole cell lysates were produced using 30 OD_600nm_ units of cell mass resuspended in 1 mL of RIPA buffer (ThermoFisher Scientific) and 3 rounds of bead beating. Cytoplasmic and membrane fractions were obtained by lysis of 30 OD_600nm_ units of cells resuspended in 1 mL 25 mM HEPES (pH 8); 200 mM NaCl. Lysate was clarified through centrifugation at 10,000 x *g*; 15 min; 4 °C. This clarified lysate was then transferred to 1 mL ultracentrifuge tubes then centrifuged at 130,000 x g; 40 min; 4 °C. The cytoplasmic fraction was then removed, and membranes resuspended in RIPA buffer to solubilise membrane proteins. Proteins were then mixed with Laemmli buffer and boiled then subjected to SDS-PAGE. Gels were then blotted using a wet transfer system and proteins detected using anti-GroEL and anti-heparin binding haemagglutinin antisera as cytoplasmic and membrane controls, respectively. N-terminally pep86-tagged EatI protein was detected using monoclonal anti-HiBit antibody (Promega; N7200; 1:3000 in Tris buffered saline). Goat anti-mouse IgG-HRP (Bio-Rad; 1706516; 1:5000 in Tris buffered saline) and chemiluminescent substrate were then used to detect bands.

### Analysis of putative T7SS-like proteins across Mycobacteriales

*M. abscessus* assemblies were downloaded from NCBI with an assembly level of scaffold or higher, packaged into local blastn (v2.13.0) and hmmer (v3.3.2) databases^65,66^. Both databases were queried under default search parameters using the WxG100 domains of known T7SSb substrates. Standard protein BLAST was used to query proteins across Mycobacteriales beyond *M. abscessus*. Candidate proteins were grouped based on C-terminal domain prediction by Motif Search using the Pfam database with default settings^67^. Topological features of corresponding immunity proteins were predicted through submission to SignalP 6.0^68^ and DeepTMHMM (v1.0.24)^69^. Genetic neighbourhoods were determined by submitting candidate proteins to the WebFlags^70^ server, with genetic variation assessed using both Cblaster^71^ and Clinker^72^ visualisation tools. Phylogenetic relationships were determined using MUSCLE alignment and constructing maximum likelihood trees with 250 bootstraps within MEGA (v10.1.8)^73,74^

### Reporting summary

Further information on research design is available in the Nature Portfolio Reporting Summary linked to this article.

## Supplementary information

Supplementary Data 1

Supplementary Information

Supplementary Software 1

Description of Additional Supplementary Files

Reporting Summary

Transparent Peer Review file

## Source data

Source Data

## Data Availability

The crystallographic data generated in this study have been deposited in the PDB under accession codes 9MXM, 9MYT, and 9MZ5. DNA sequencing results are deposited in the European Nucleotide Archive under project PRJEB85598. All AlphaFold 3 models are available in Supplementary Data 1. The remaining data generated in this study are provided in the Supplementary Information and Source Data file. Source data are provided with this paper.

## Code availability

The R script used for fluorescence data analysis is available as Supplementary Software 1 and at GitHub (https://github.com/Hudaahmadd/Fluorescence-Analysis).
