# Supplementary material for: A Type VII-secreted toxin enables inter-mycobacterial competition
Source: Nat Commun. 2026 Jun 23;17:7899. doi: 10.1038/s41467-026-74429-7 (PMC13444075; doi:10.1038/s41467-026-74429-7)
Supplement: Supplementary file 1 [file 41467_2026_74429_MOESM2_ESM.pdf]

Supplementary Materials for:

A Type VII-secreted toxin enables inter-mycobacterial competition

Samuel T. Benedict[1,2], Kieran Bowran[2], Eunice K.E. Lee[2], Jean-Lou Reyre[2], Huda Ahmad[1], Aaron Franklin[1], Nicole A. Mietrach[2], Cheng-Ruei Han[3], Jia Mun Chan[2], Abigail Layton[1], Emmanuele Severi[2,4], Eleanor R. Boardman[2], Kamilla Anochshenko[1], Gregory Goudge[1], Simon G. Caulton[1], Todd L. Lowary[3,5], Andrew L. Lovering[1], Manuel Banzhaf[2], Elisabeth C. Lowe[2], Tracy Palmer[2‡], Patrick J. Moynihan[1,6‡]

‡ Co-corresponding author; pmoyniha@uwo.ca or tracy.palmer@newcastle.ac.uk

Affiliation:

1. School of Biosciences, University of Birmingham, Birmingham, U.K., B15 2TT
2. Newcastle University Biosciences Institute, Medical School, Newcastle University, Newcastle upon Tyne, U.K., NE2 4HH
3. Institute of Biological Chemistry, Academia Sinica, Nangang, Taipei, Taiwan
4. Northumbria University, School of Geography and Natural Sciences, NE1 8ST Newcastle upon Tyne.
5. Institute of Biochemical Sciences, National Taiwan University, Taipei, Taiwan
6. Department of Microbiology and Immunology, Schulich School of Medicine and Dentistry, The University of Western Ontario, London, Ontario, Canada

Supplementary Figure 1. Type VIIa and VIIb secretion motifs are conserved across diverse substrates.

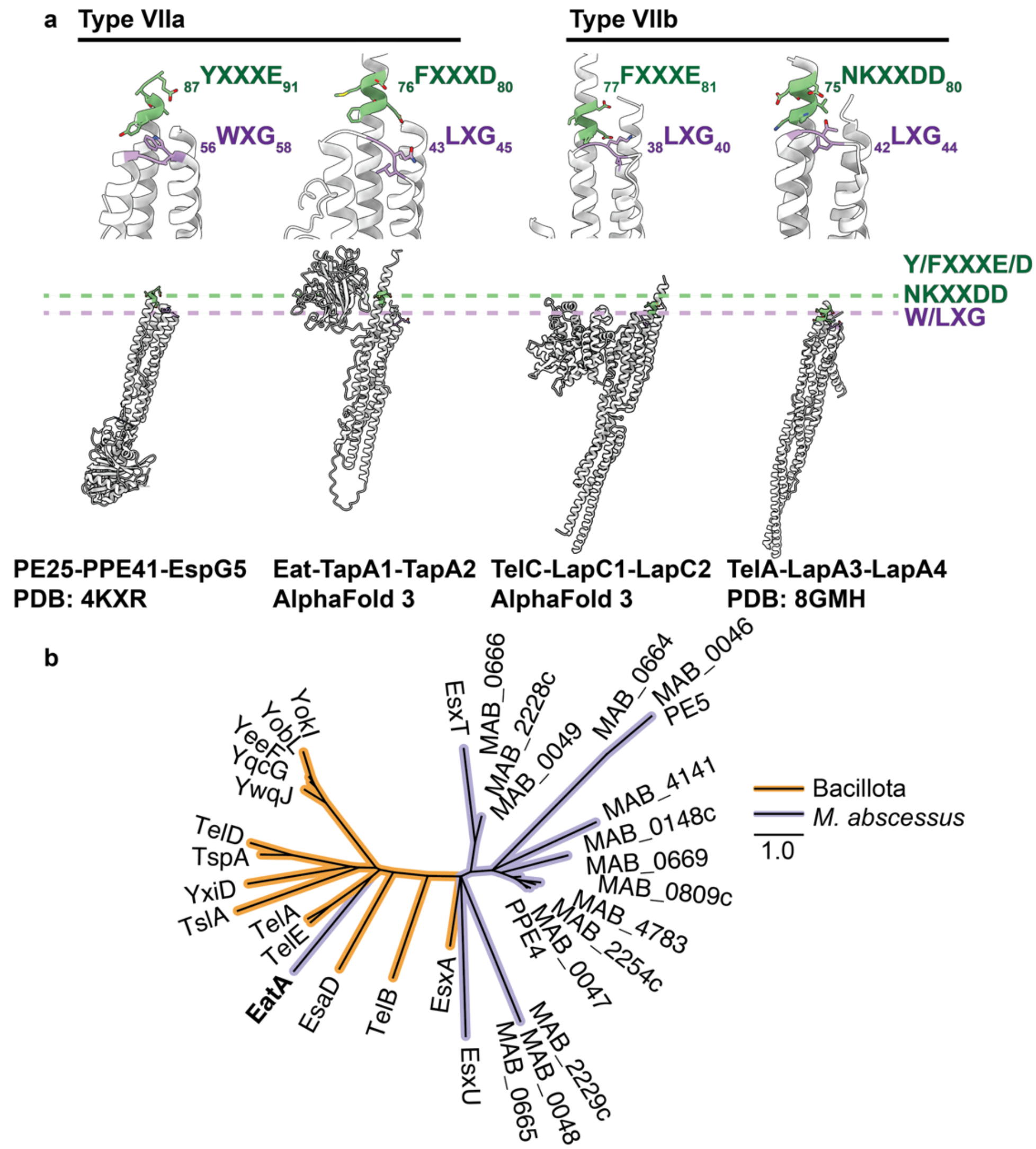

a, Across both Type VIIa and VIIb secretion systems, two motifs have been experimentally determined to be essential for cargo secretion. These are the W/LXG and Y/FXXXE/D (or NKXXDD) (upper panel). Alignment of the AlphaFold 3 (prediction statistics in Supplementary Fig. 2) or experimentally determined structures for EatA-TapA1-TapA2, TelC-LapC1-LapC2 and TelA-LapA3-LapA4 complexes to the experimentally determined structure of PE-25-PPE41-EspG5 demonstrates conservation of these motifs across Type VII substrates (lower panel). The dashed lines indicate the location of the motifs in the individual structures. AlphaFold statistics are presented in Supplementary Fig. 2. **b,** Phylogenetic analysis of the N-terminal helical domain of T7 substrates from the Bacillota (orange) and *M. abscessus* ATCC 19977. Protein sequences for all proteins annotated as PE or PPE and selected Bacillota T7b substrates were chosen for analysis. This was restricted to the N-terminal 200 (TelA-E, YxiD, TspA, YwQJ, YqcG, YeeF, YobL, YokL, EsaD, EatA, and MAB_4141, 0148, 0669, 0809c, 4783, 2254c, 0047, PPE4), 97 (EsxA, EsxT, MAB_2229c, 0048, 0665), 96 (MAB_0666, MAB_2228c), 102 (MAB_0664, 0046, PE5), 92 (EsxU) and C-terminal 199 (TslA) amino acids to capture the helical bundle domain.

Supplementary Figure 2. AlphaFold3 prediction of EatA-TapA1-TapA2 and TelC-:LapC1-LapC2 complexes.

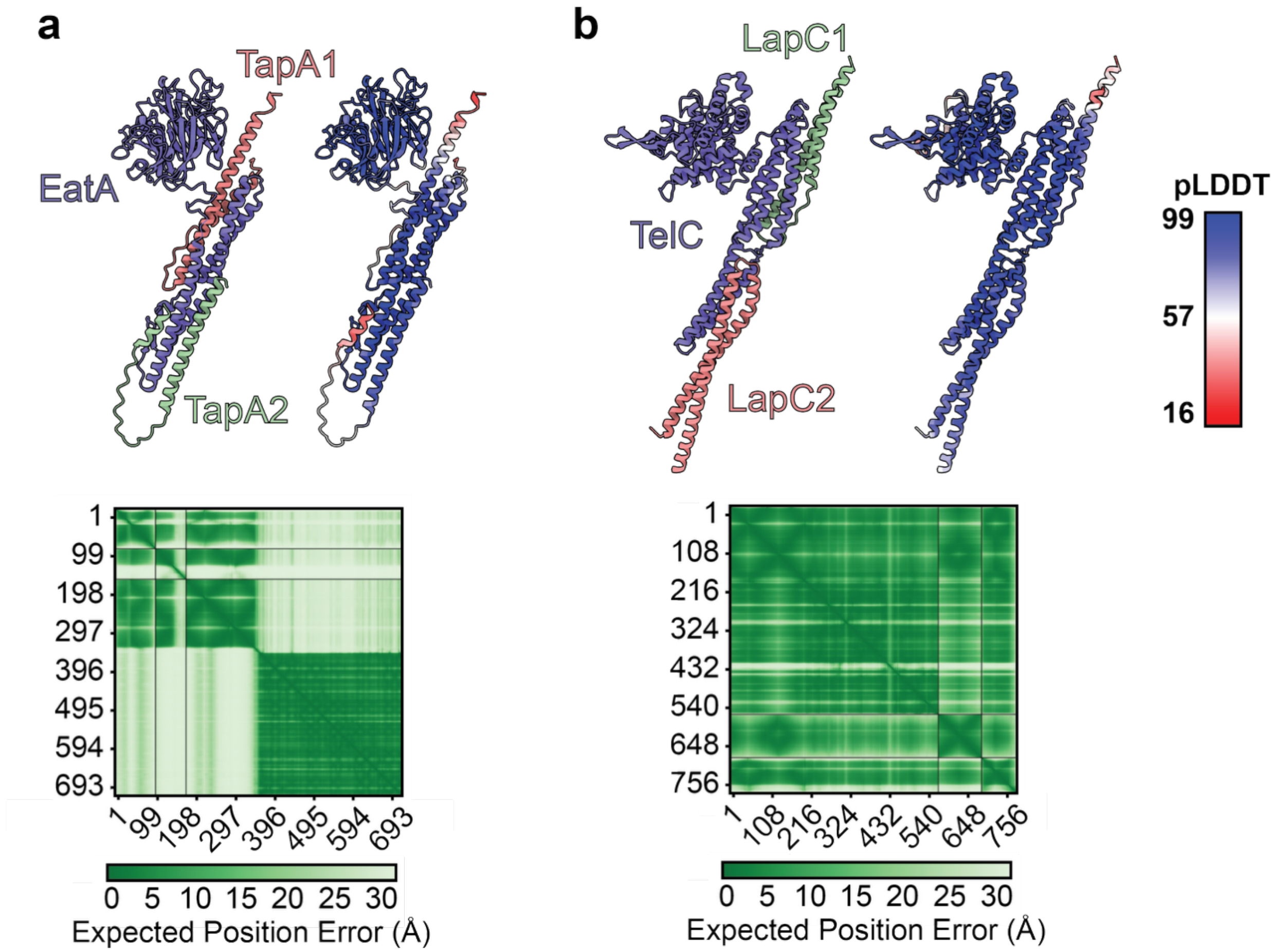

The amino acid sequences for **(a)** EatA, TapA1 and TapA2 (MYCMA_14050/55/60) and **(b)** TelC-LapC1-LapC2 (SIR_1489/90/91) were inputted to the AlphaFold 3 server using a single copy of each protein. For each protein, the predicted structure is shown on the left, coloured by chain, and on the right, according to per-atom pLDDT with a scale to the right of the panels. Underneath each image is the expected position error plot produced by AlphaFold 3 with the predictions.

Supplementary Figure 3. EatA enzymes are inhibited by cognate EatI proteins

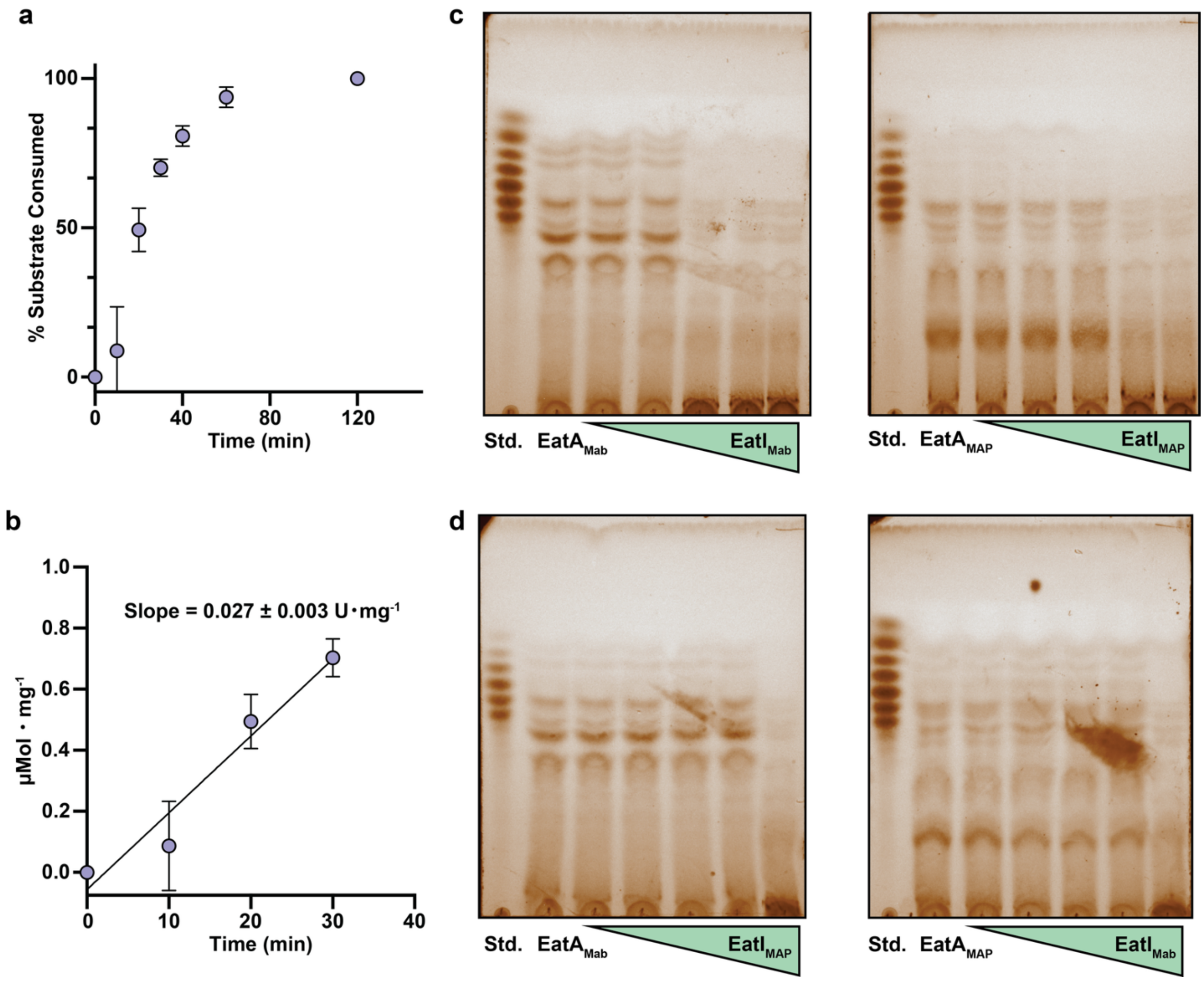

a, Substrate peaks from Fig. 2a were integrated and the percentage of substrate consumption relative to the amount of substrate at 0 min is reported. Error bars represent the standard deviation of three biological replicates. **b,** The linear portion of the curve in **a** was converted to µMol substrate conversion by comparing to standards of known concentration. A linear regression was computed in Graphpad Prism 10.4.1. Error bars represent the standard deviation of three biological replicates. $EatA_{Mab}$ and $EatA_{MAP}$ were incubated with their cognate (**c**) or non-cognate (**d**) EatI before initiating reactions with $1\ mg \cdot mL^{-1}$ arabinogalactan from *M. smegmatis* mc^2155. Reactions were carried out at 0:1, 0.25:1, 0.5:1, 1:1, 2:1 and 2:0 EatI:EatA molar ratios for 30 min at 37 °C before being stopped by boiling, separated by TLC two times in a solvent system of 2:1:1 butanol:acetic acid:water and visualised by staining with orcinol and charring. Representative data from two biological replicates are presented. TLCs have been false-coloured in ImageLab 6.1 after black-and-white imaging.

Supplementary Figure 4. Mass spectrometry analysis of Eat reaction products.

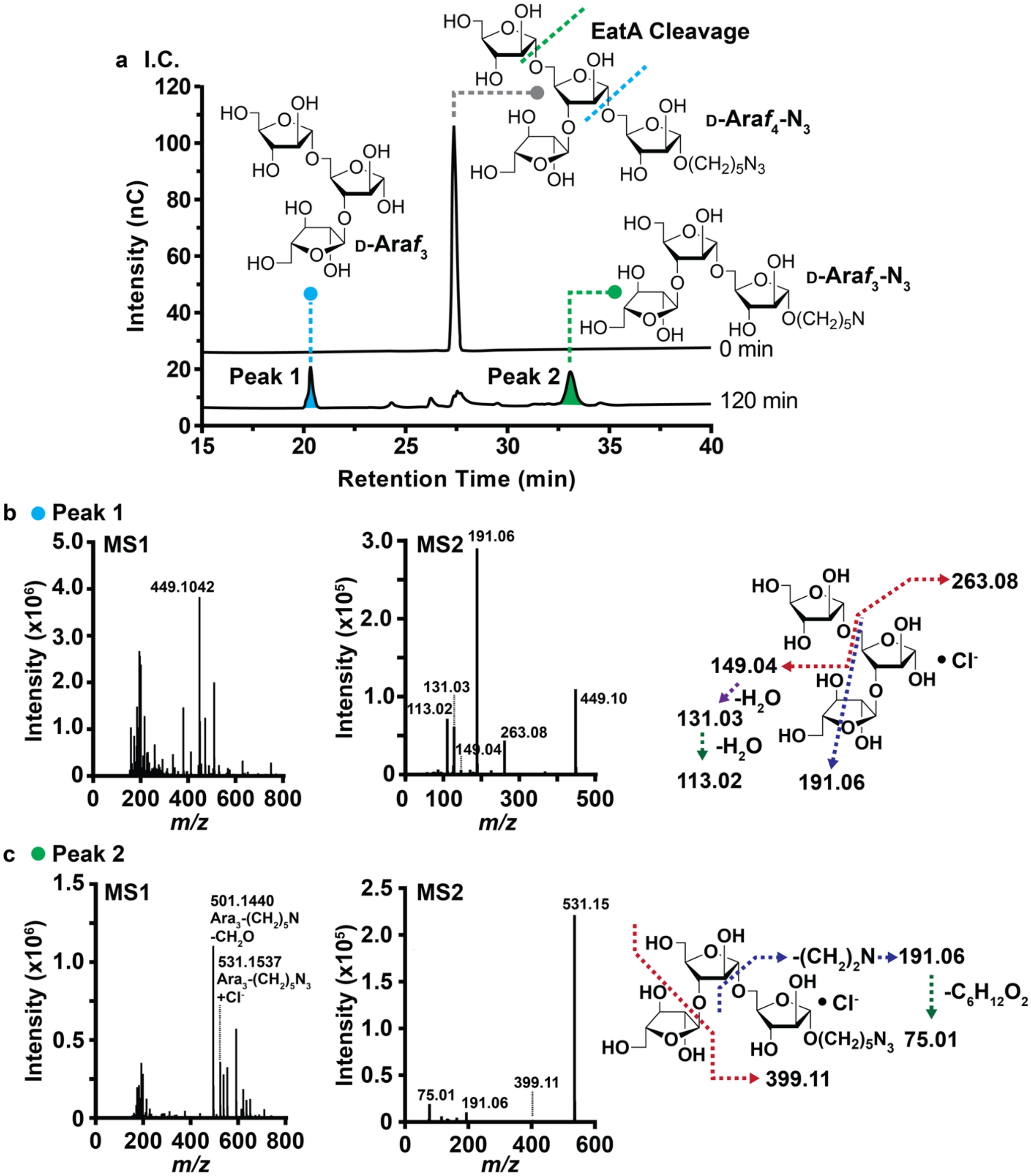

The reaction described in Fig. 2a was subjected to ion-chromatography-mass spectrometry to determine the structure of the reaction products. For each peak, the major Cl^- adduct in the MS1 was chosen for MS2 analysis due to in-source decay of other ions. **a,** The fragment observed in peak 1 is consistent with a D-arabinofuranose (D-araF) trimer, which has been cleaved from the arabinose attached to the azide-containing linker via cleavage of the 1,5 glycosidic linkage. **b**, The fragment observed in peak 2 is consistent with cleavage of the terminal 1,5 linked arabinofuranose unit, again via cleavage of the 1,5 glycosidic linkage.

Supplementary Figure 5. Analytical size exclusion of EatA-EatI complexes.

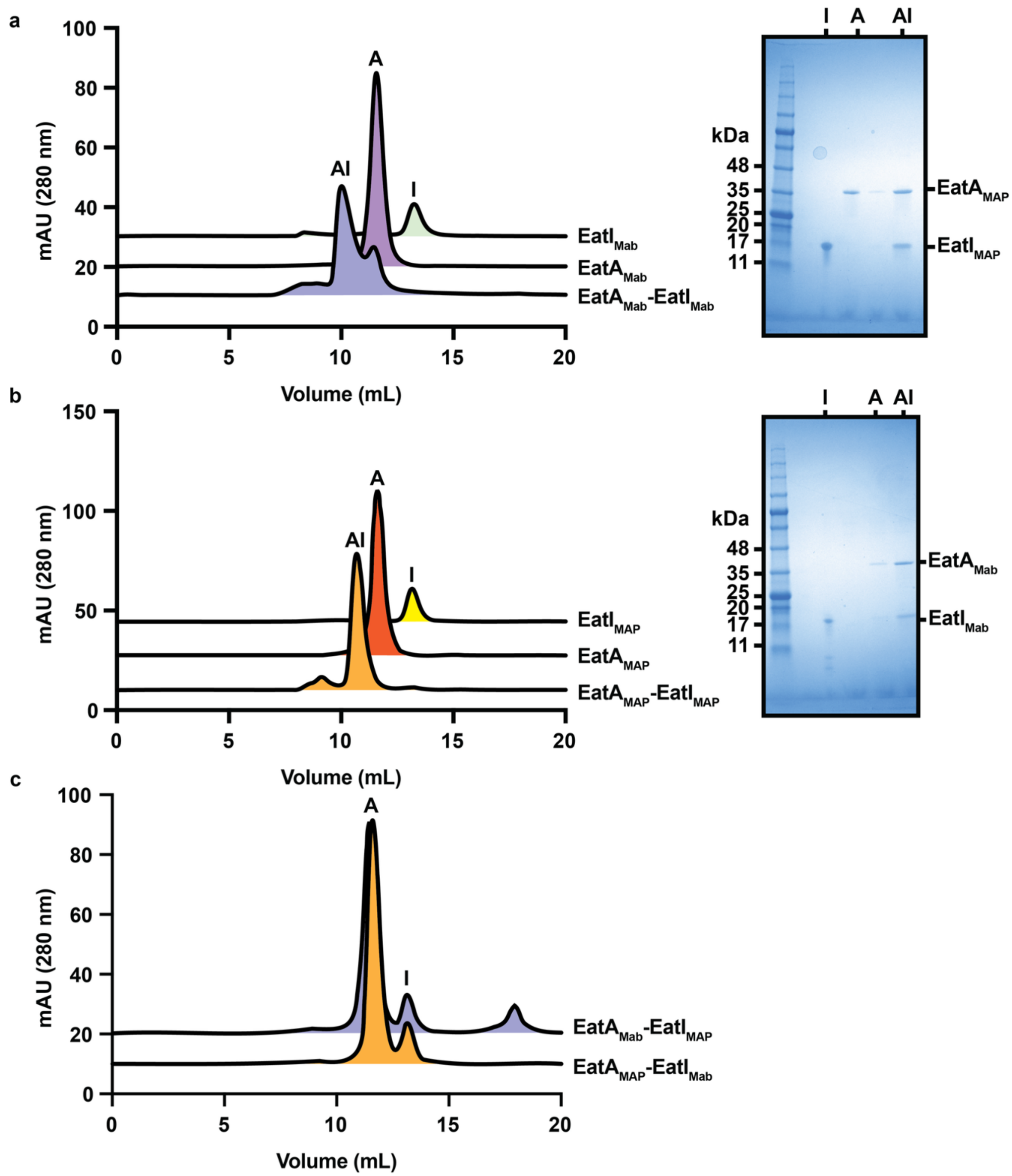

Recombinantly produced EatA GH183 domains and EatI proteins from *M. abscessus* (**a**) and *M. avium* subsp. *paratuberculosis* (**b**) were incubated alone or in combination and then analysed by size-exclusion chromatography on a Superdex 75 Increase 10/300 GL column. To identify peak fractions of the isolated proteins, higher molecular weight complexes were collected and analysed by SDS-PAGE (right). **c,** Non-cognate pairs of EatA and EatI were analysed similarly, demonstrating a lack of complex formation. The low molecular weight peak at 17.5 mL in EatI$_{Mab}$-EatA$_{MAP}$ is a contaminating protein. I – EatI, A – EatA, AI – EatA + EatI.

Supplementary Figure 6. Structural analysis of substrate and immunity interaction interfaces in EatA proteins.

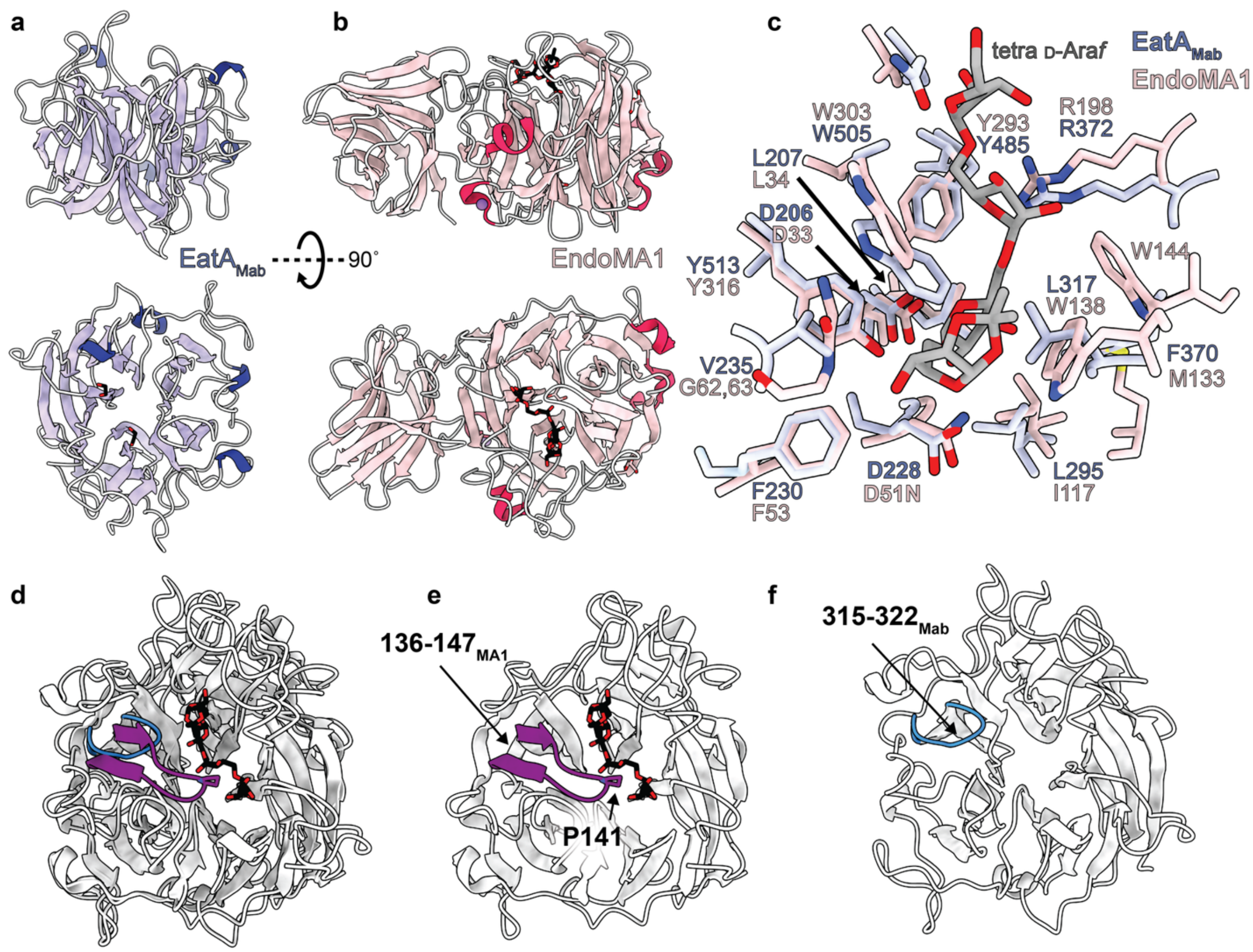

Aligned (RMSD = 5.48 Å) top and side views of EatA$_{Mab}$ (**a**) and EndoMA1 (**b;** PDB:8IC1). **c,** Key binding and catalytic (EatA$_{Mab}$ - D206, D228) residues are conserved between EndoMA1 and EatA$_{Mab}$. **d,** Comparison of the aligned cartoon representation of EndoMA1 highlighting the loop containing P141 (EndoMA1 A.A.s 136-147; purple) and the equivalent loop in EatA$_{Mab}$ (A.A.s 315-322; blue). **e,** EndoMA1 in isolation from panel d. **f,** EatA$_{Mab}$ in isolation from panel d. Ligands derived from crystallization conditions (K^+, ethylene glycol) have been omitted from the figure to aid visualization.

Supplementary Figure 7. Structural basis for absence of non-cognate inhibition by EatI.

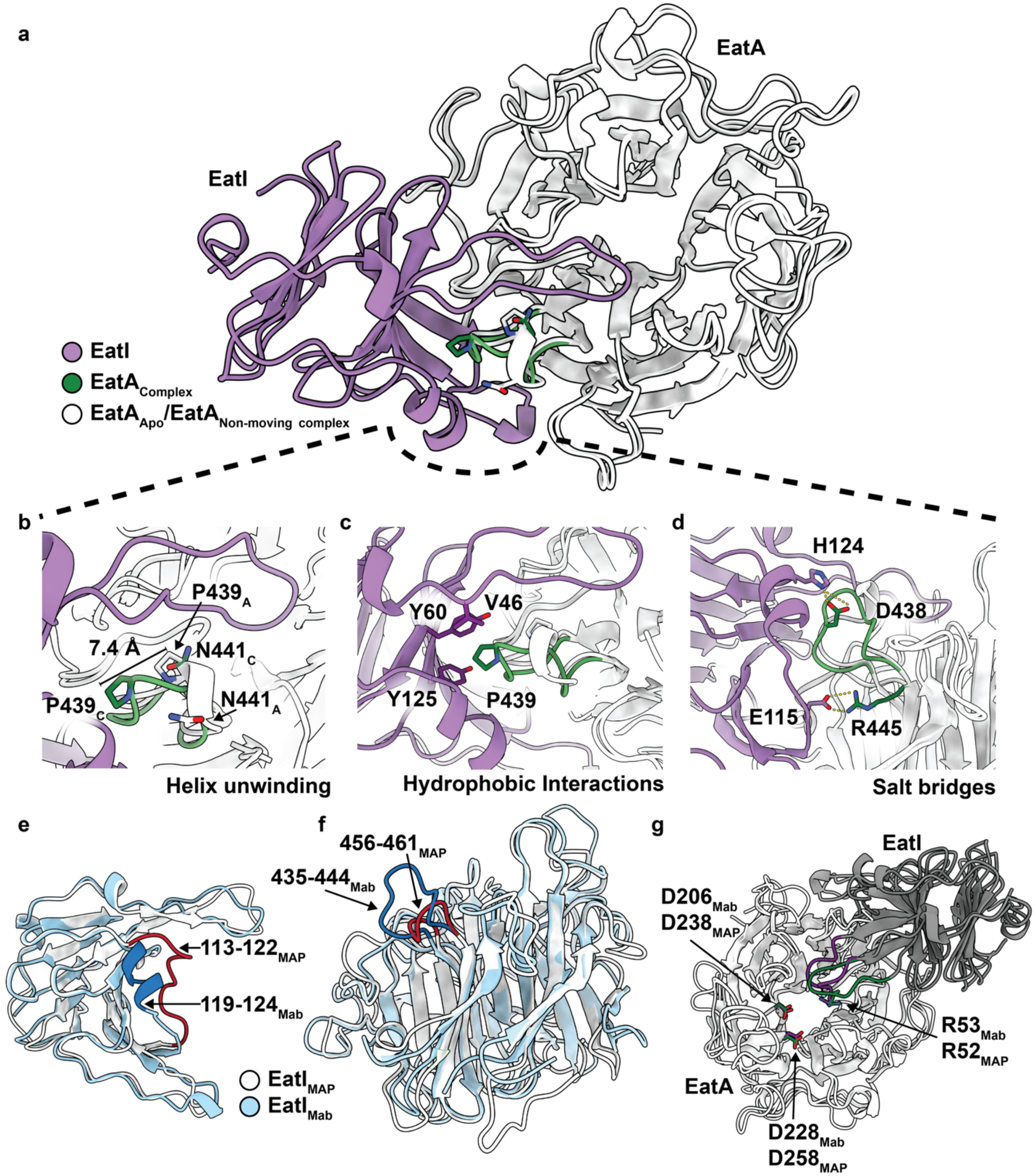

a, Structural alignment of $EatA_{Mab\text{-}Apo}$ and $EatA_{Mab\text{-}Complex}$. The key interface motif is highlighted green in the $EatA_{Mab\text{-}Complex}$ structure. **b,** Binding of EatA triggers an unwinding of a small helical turn (439-441; white in $EatA_{Mab\text{-}Apo}$; green in $EatA_{Mab\text{-}Complex}$), moving P439 7.4 Å towards $EatI_{Mab}$ (presented in purple). **c,** This movement creates hydrophobic interactions between P439 in

EatA$_{Mab}$ with V46, Y60 and Y125 in EatI$_{Mab}$. **d,** This is accompanied by the formation of salt bridges between EatI H124 and EatA D438 and EatI E115 and EatA R445. **e,** When aligned, the EatA-EatI$_{Mab}$ and EatA-EatI$_{MAP}$ structures have an overall RMSD of 2.6 Å. When viewed in isolation, the EatI$_{Mab}$ helix formed by residues 119-124 (blue) is instead a loop moved laterally formed of resides 113-122 (red) in EatI$_{MAP}$. **f,** This change is accompanied by alterations in the binding surface on EatA, most notably a truncation of the EatA$_{Mab}$ interface loop (435-444; blue) in EatI$_{MAP}$ (456-461; red). **g,** Despite these interface changes, when the structures are aligned based on EatA, a conserved EatI Arg (R53$_{Mab}$/R52$_{MAP}$) residue is coordinated in equivalent positions in the active sites in the EatA-EatI$_{Mab}$ and EatA-EatI$_{MAP}$ complexes.

Supplementary Figure 8. An *M. abscessus* ATCC19977 Δ*eccC4* mutant no longer secretes EsxU.

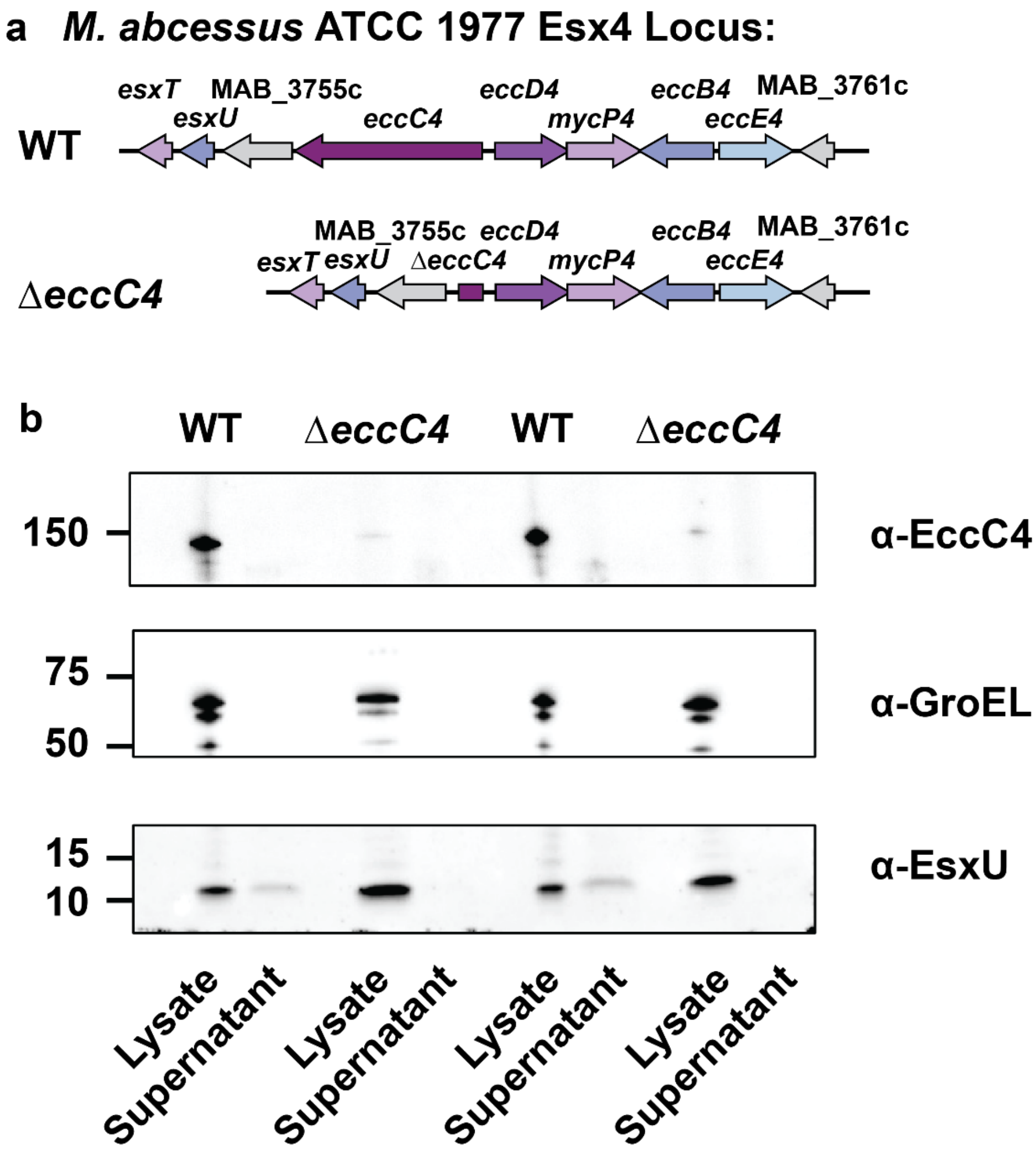

a, The *M. abscessus* ATCC 19977 *esx-4* locus in the wild-type and Δ*eccC4* mutant background. **b,** This mutation leads to a loss of function for ESX-4 as evidenced by the loss of EsxU secretion in the mutant culture supernatant. Left and right are biological replicates.

Supplementary Figure 9. Expression of *eatI* from a strong promoter is toxic.

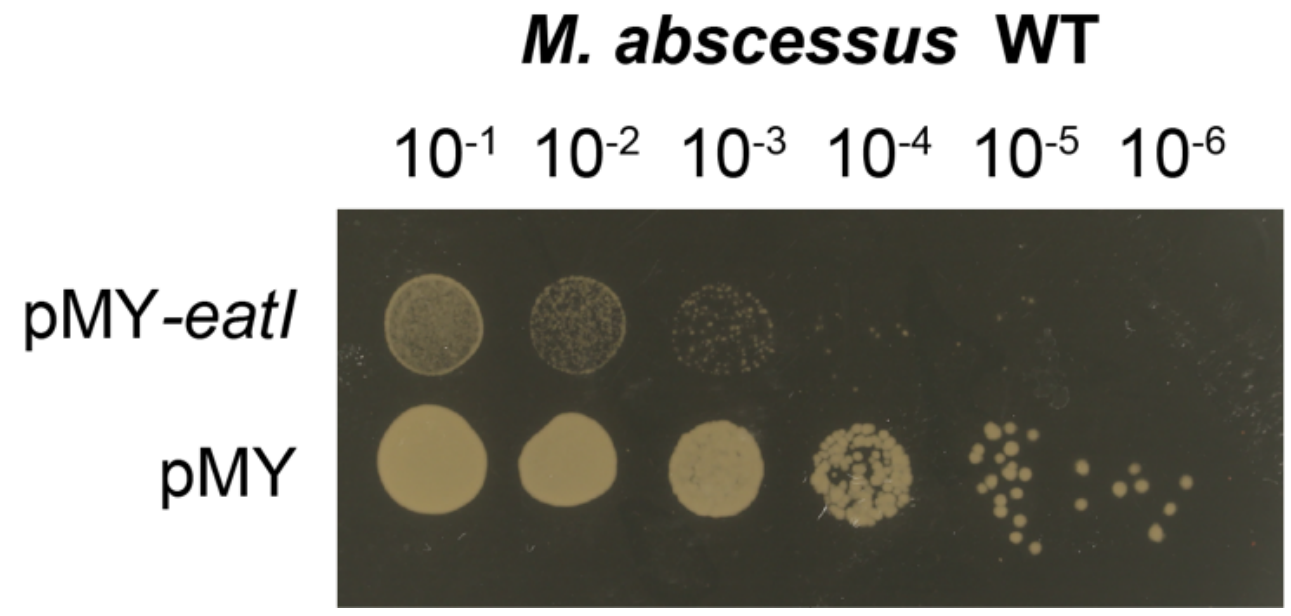

M. abscessus ATCC 19977 was transformed with either an empty acetamide inducible pMY plasmid (pMY) or the same backbone containing $eatI_{Mab}$. Cultures were grown to OD_{600nm}= 0.6 and then serially diluted and spotted on to tryptic soy agar. Plates were imaged after the appearance of colonies.

Supplementary Figure 10. Colony size statistics for self-intoxication in *M. abscessus*

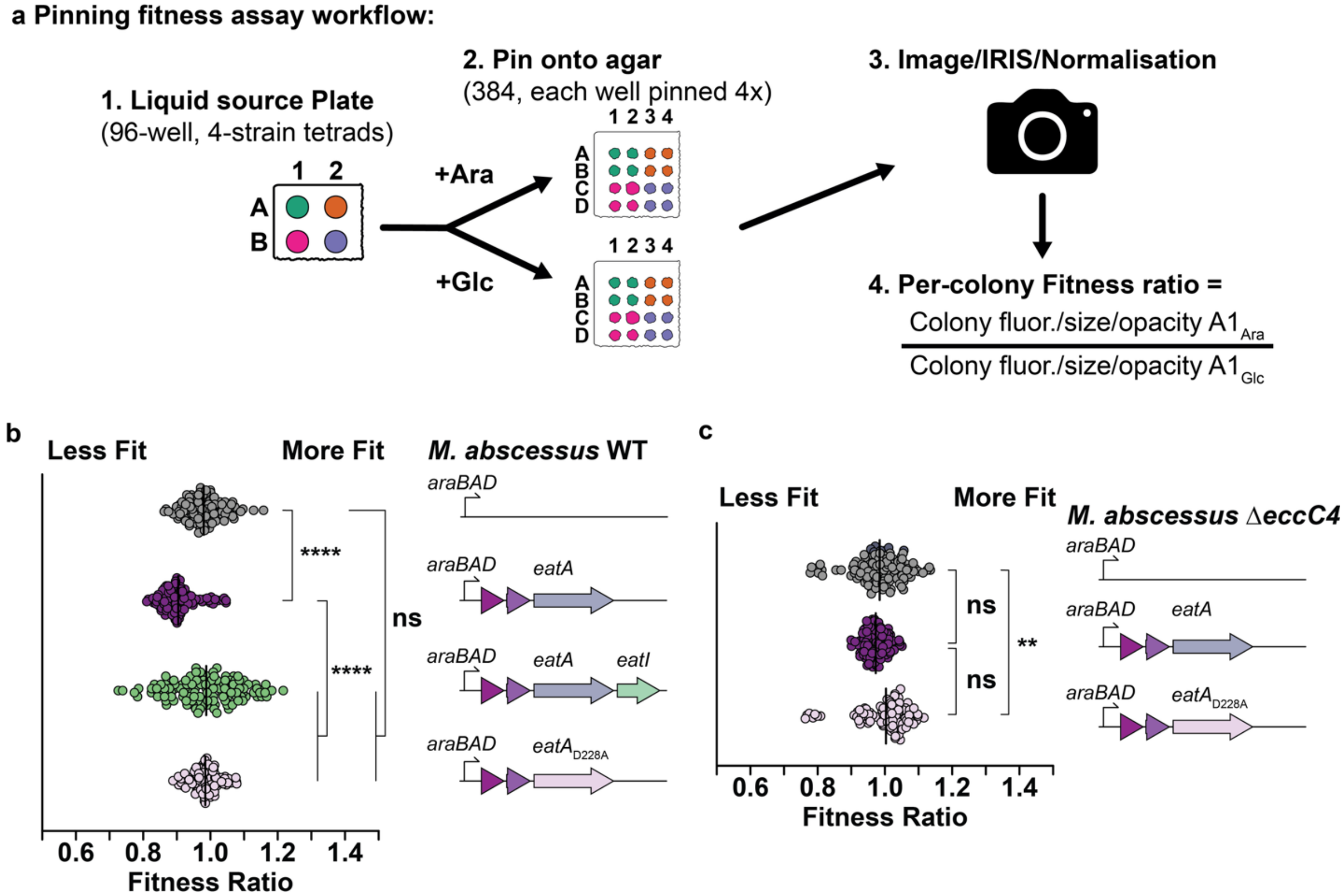

a, Workflow for pinning assays. (1.) A 96-well source plate (2 mL) of culture is arrayed, and then (2.) pinned in quadruplicate onto solid agar, giving 384 total colonies. (3.) These are imaged and processed as described in the methods. (4.) Finally, a fitness ratio is calculated by dividing the colony size/opacity or fluorescence of the same colony pinned on glucose or arabinose. *M. abscessus* ATCC 19977 **b,** or a Δ*eccC4* derivative **c,** were transformed with a pMYBAD plasmid containing the indicated components of the *eatA* locus under the control of an arabinose inducible promoter. These were pinned (n = 128) onto tryptic soy agar with either 1% D-glucose or L-arabinose, grown for 48 h and imaged. Fitness is measured as a ratio of the colony size grown in inducing (L-arabinose) divided by non-inducing (D-glucose) conditions. Raw colony images presented in Supplementary Fig. 11.

Supplementary Figure 11. Pinning images

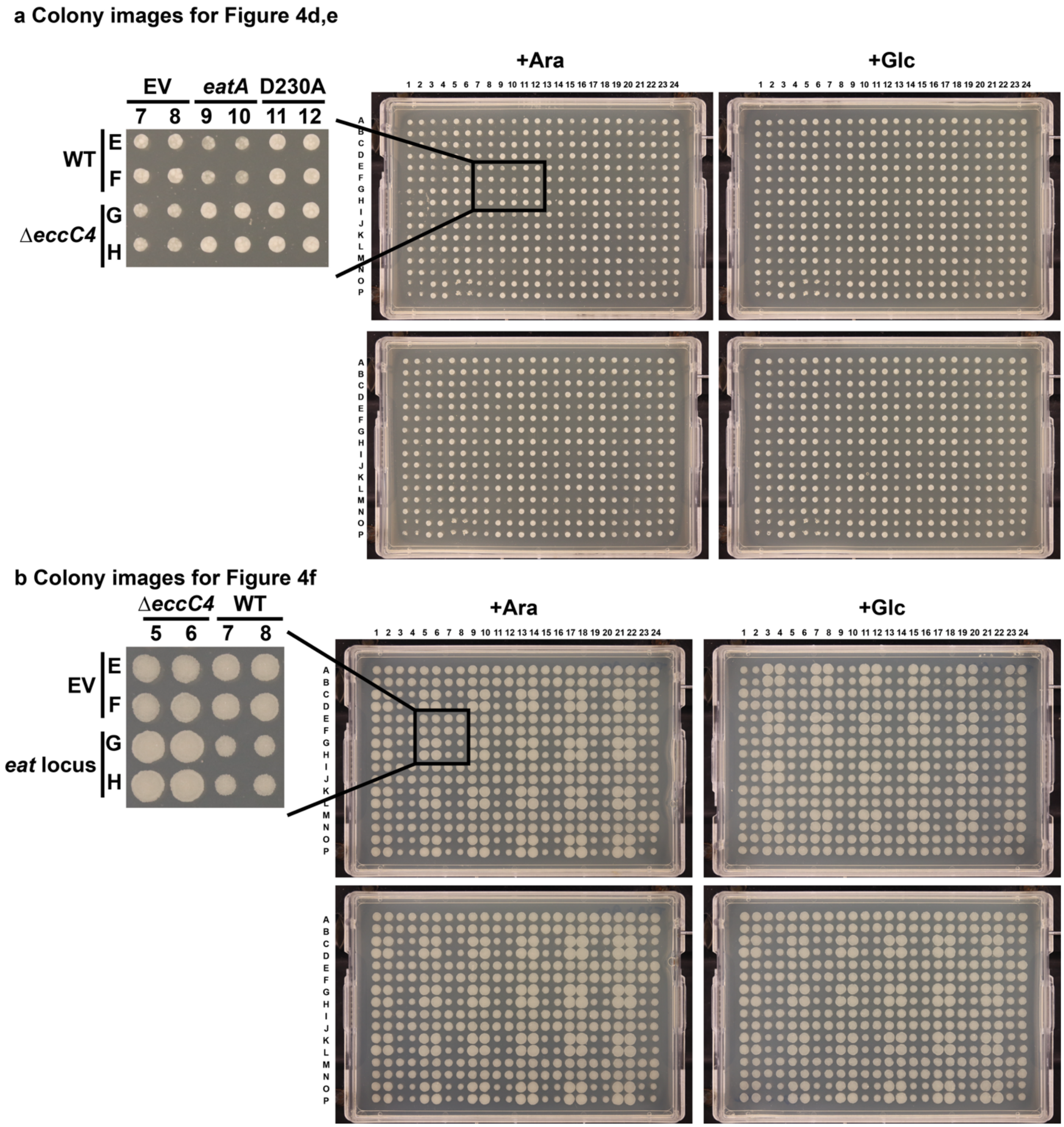

a, Raw colony images for Fig. 4d,e, Supplementary Fig. 10b. **b,** Raw colony images for Fig. 4f.

Supplementary Figure 12. ***M. smegmatis*** **fluorescence correlates with culture OD_{600nm}**

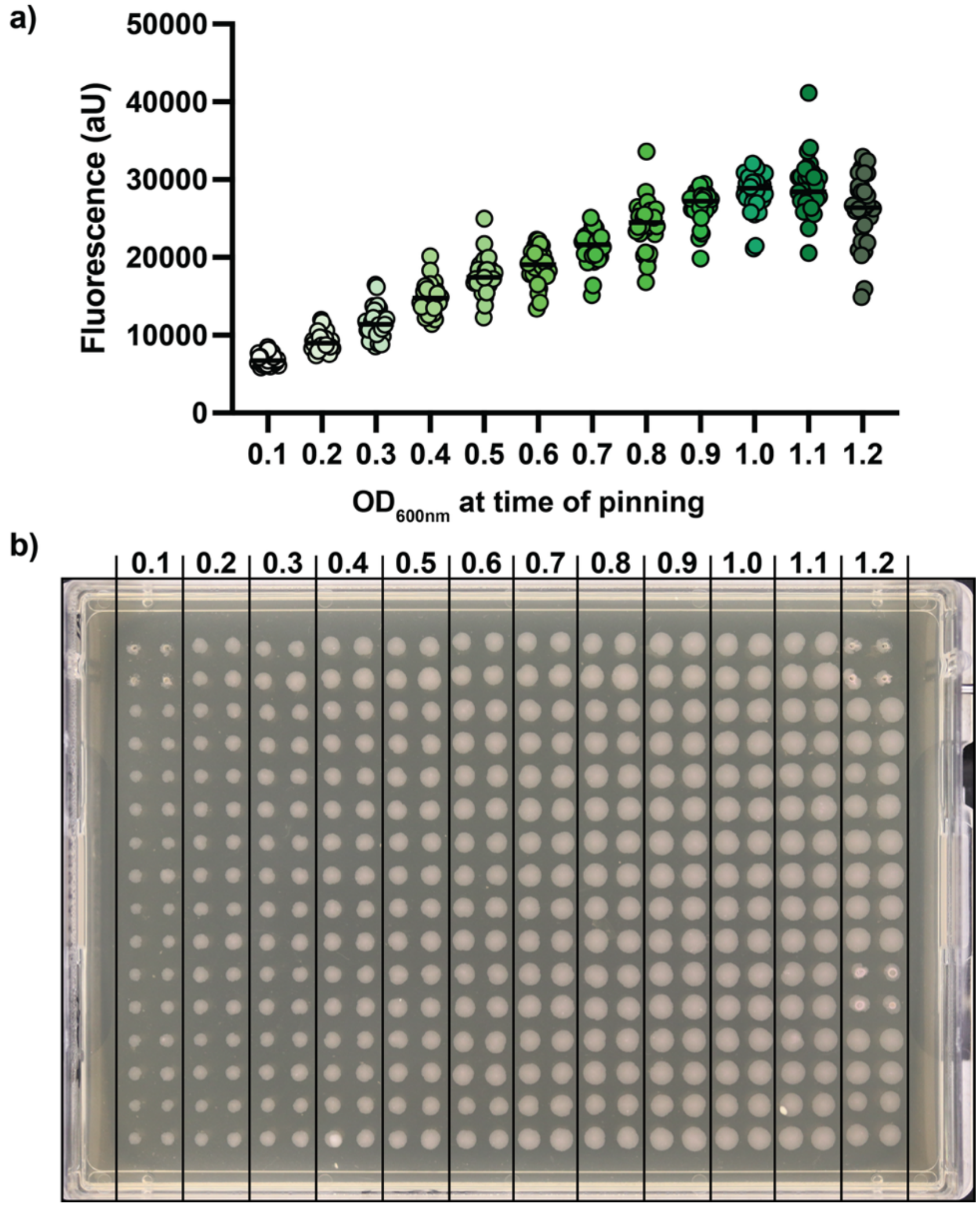

M. smegmatis mc²155 pTEC27-tdTomato was prepared at defined starting optical densities (OD_{600nm} = 0.1–1.2) in a 96-well plate, with each column containing a single OD and 12 replicates per condition. Cultures were pinned from the source plate onto 7H9 and TSB agar using a S&P robotics robotic pinning system, with each source well pinned four times (n = 48 replicate spots per starting OD_{600nm}). Plates were incubated at 37 °C and imaged using a plate reader to measure fluorescence (Ex = 544, Em = 590 nm, Gain = 2000). Colony size was quantified using Iris software (version 0.9.7; https://github.com/critichu/Iris). Fluorescence and colony size measurements were analysed in R. In both analyses, strain effects were assessed using one-way ANOVA followed by Tukey's HSD post-hoc tests, and statistical significance was visualized using swarm plots with adjusted *p*-values. Notably, all pair-wise comparisons were at least $p < 0.005$, though above culture OD of 1.0 the correlation reversed whereby fluorescence appears to decrease with increasing colony size. Source code for the analysis is available on GitHub at https://github.com/Hudaahmadd/flu-size-qc. n = 32, except for OD_{600nm} 1.2 where 8 colonies were rejected due to air bubbles.

Supplementary Figure 13. Toxin-like loci in *Mycobacterium tuberculosis* lineages

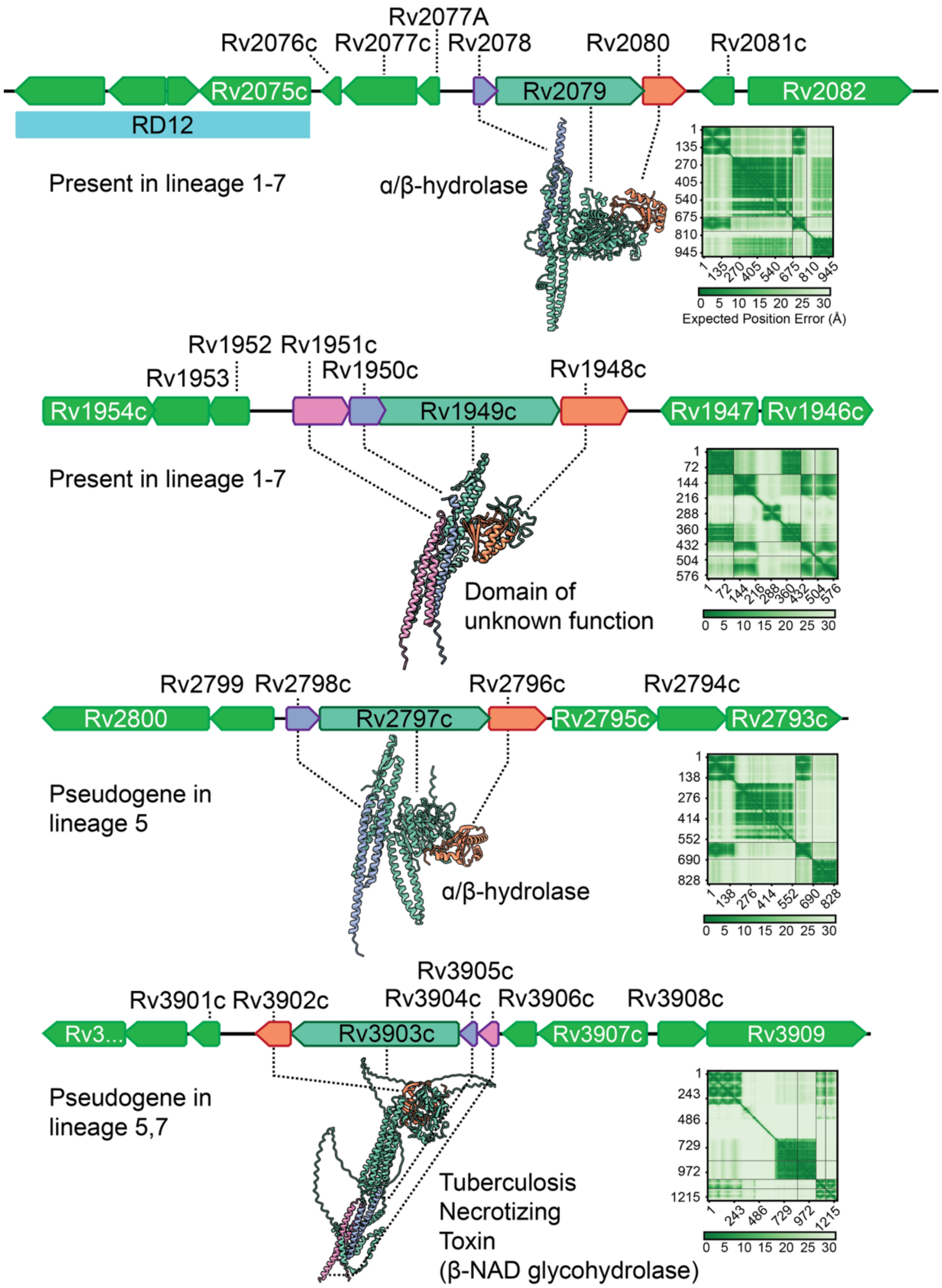

Representative genomes from the 7 major *M. tuberculosis* genomes were interrogated for toxin-like loci using BLAST, AlphaFold 3.0 and Foldseek, revealing 4 high-quality hits. These have a consistent locus structure (H37Rv loci shown), and the Alphafold prediction is consistent with other T7Ssa substrates. One of these is tuberculosis necrotizing toxin. Alphafold 3.0 models are coloured according to the locus diagram and the expected position error plots are presented to the right. (Representative strains: N0069 (L1), N0031 (L2), N0004 (L3), N0136 (L4), N1176 (L5), N0091 (L6), N3913 (L7), H37Rv).

Supplementary Figure 14. Uncropped gels, blots and TLCs.

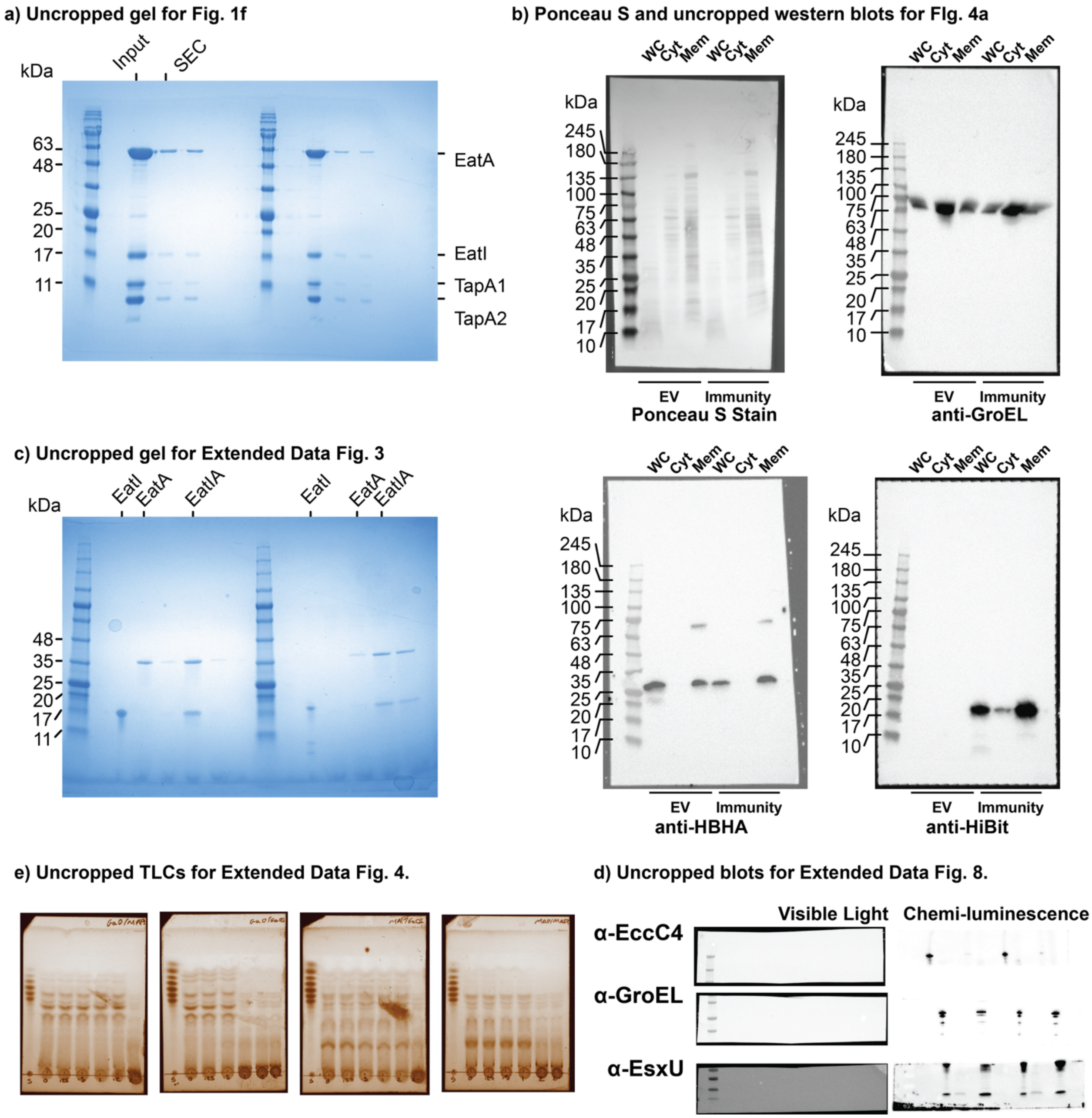

Uncropped gels for Figures **(a)** 1f **(b)** 4b, **(c)** Supplementary Fig. 3, **(d)** Supplementary Fig. 4., **(e)** Supplementary Fig. 8.

Supplementary Figure 15. Example density for X-ray crystal structures

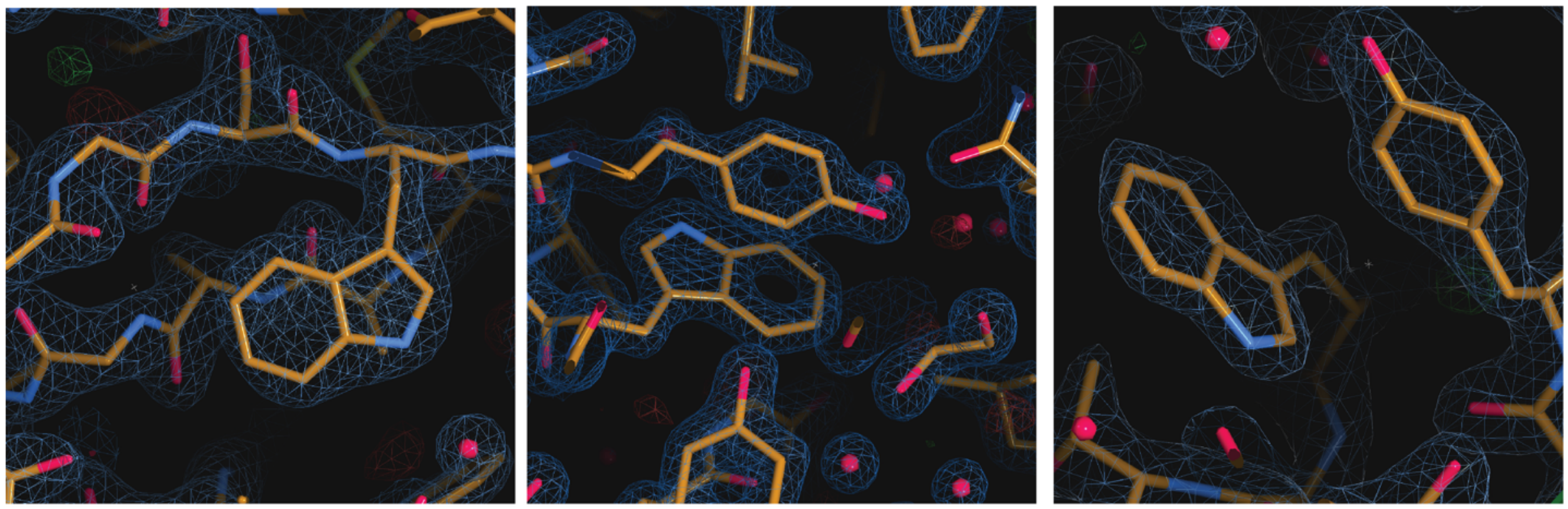

EatA$_{Mab}$ Apo **EatA-I$_{Mab}$** **EatA-I$_{MAP}$**

The map and model for 9MXM (EatA$_{Mab}$-Apo), 9MYT (EatA-I$_{Mab}$)and 9MZ5 (EatA-I$_{MAP}$) were opened in Coot and the n-rmsd set to 2.0. Panels show representative areas of the structures.

Supplementary Figure 16: pLDDT of Alphafold models

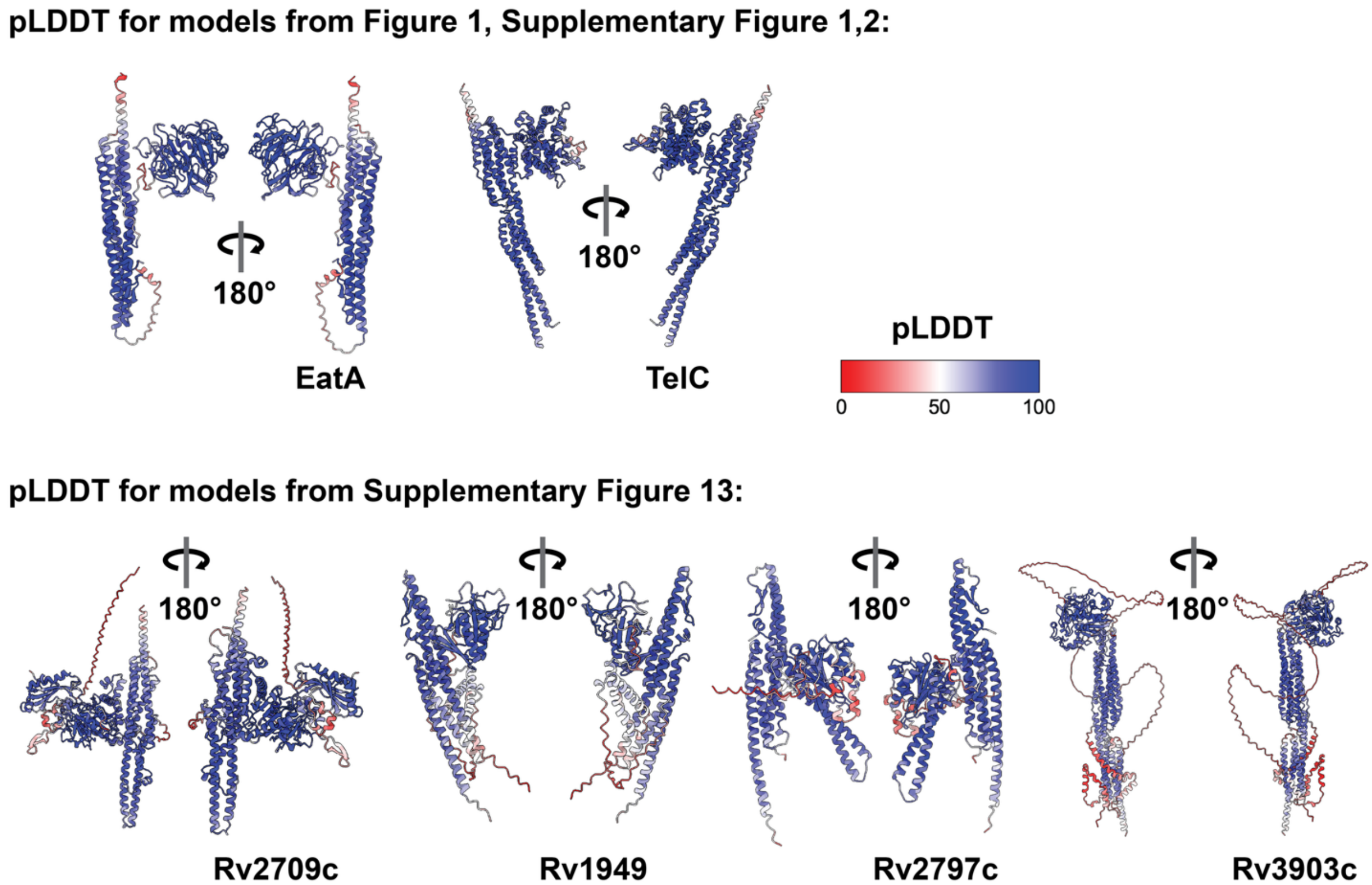

Alphafold models were generated using the Alphafold 3 server (alphafoldserver.com). Models were opened in ChimeraX and coloured by per-residue pLDDT with a red to blue gradient as indicated in the key above.

Supplementary Table 1. Crystallography statistics.

	EatAMab	EatA-EatIMab	EatA-EatIMAP
**PDB Code:**	9MXM	9MYT	9MZ5
**Collection Statistics:**			
Wavelength	0.9795 Å	0.96863 Å	0.6199 Å
Resolution range	51.97 - 2.06 (2.1 - 2.06)	89.917 - 1.702 (1.732 - 1.702)	77.009 - 1.650 (1.792 - 1.650)
Space group	$C222_1$	$P2_1$	$P2_12_12_1$
Unit cell	87.96 94.79 175.64 90 90 90	51.584 99.521 92.092 90 102.48 90	67.26 101.469 118.264 90 90 90
Total reflections	307785	575884	773467
Unique reflections	45733	89875	68287
Multiplicity	6.7 (7.1)	6.4 (4.9)	11.3 (6.6)
Completeness (%)	100 (100)	90.6 (49.2)	88.5 (47.7)
Mean I/sigma(I)	5.5 (0.8)	6.3 (0.5)	10.9 (1.6)
R-merge	0.224 (2.177)	0.137 (2.223)	0.140 (1.078)
R-meas	0.244(2.35)	0.150 (2.482)	0.146 (1.167)
R-pim	0.095 (0.88)	0.059 (1.069)	0.041 (0.432)
CC1/2	0.989 (0.312)	0.989 (0.37)	0.998 (0.717)
**Refinement Statistics**			
Reflections used in refinement	45680	89289	68264
Reflections used for R-free	2325	4238	3508
R-work	0.1922	0.1574	0.1601
R-free	0.2328	0.1973	0.1942
Number of non-hydrogen atoms	5481	8415	8053
macromolecules	5051	7309	6966
ligands	41	93	32
solvent	389	1013	1055
Protein residues	685	976	935
RMS(bonds)	0.014	0.008	0.010
RMS(angles)	1.22	0.93	1.09
Ramachandran favored (%)	95.74	97.42	97.51
Ramachandran allowed (%)	4.26	2.58	2.38
Ramachandran outliers (%)	0	0	0.11
Rotamer outliers (%)	0.56	0.38	0.53
Clashscore	5.84	3.09	5.79
Average B-factor	44.29	30.87	21.45
macromolecules	44.09	29.70	20.27
ligands	58.02	39.29	29.43
solvent	45.53	38.57	28.97

Supplementary Table 2. Primers used in this study.

Construct	Oligonucleotides for cloning	
pqe70-*eatI*-*tapA1*-*tapA2*-TS-*eatA*-His$_6$	backbone_F	catcaccatcaccatcactaagct
	backbone_R	catgcttaatttctcctctttaatgaattctgtgtg
	A1_F	atttcacacagaattcattaaagaggagaaattaagcatggcctgcaacgcatccg
	A1_R	ctcctgatcccggtcagaatctatatcttgggaatccagccggaac
	A2_F	gctggattcccaagatatagattctgaccgggatcaggagg
	A2_R	tgtggatgactccaagcactcatattctgtgggccggagg
	A3_F	cctccggcccacagaatatgagtgcttggagtcatccacaattc
	A3_R	tacatattctgtgggccggactacttctcaaattgtggatgagaccag
	A4_F	atccacaatttgagaagtagtccggcccacagaatatgtaggc
	A4_R	caagctcagctaattaagcttagtgatggtgatggtgatgttgatgtgggtttacttcgatcaattggg
pET28aEatA$_{Mab}$ D228A	D228AF	ggtttttggtgcgagcttcagcac
	D228AR	gctaccagttttttgcc
pMYBAD-*eat*	D228AF	tgtctttggagcgtctttcagcac
	D228AR	gcgacaagcttcttac
pUT18		ttgcatgcctgcaggtcg
pUT18_*tapA1*		tgaccatgattacgccaagcagtgatgaacaggagctac
		gtcgacctgcaggcatgcaagcggaacctctggaattg
pUT18_*tapA2*		tgaccatgattacgccaagcggattgaacatagatcttgatcgtg
		gtcgacctgcaggcatgcaacatattctgtgggccggag
pUT18_*eatA*		tgaccatgattacgccaagcccgacgaaatcagaaattc
		gtcgacctgcaggcatgcaattgatgtgggtttacttc
pUT18_*eatA*_ N-terminus		tgaccatgattacgccaagcccgacgaaatcagaaattcttaattg
		gtcgacctgcaggcatgcaatcgatggccactgaagtc
pUT18_*eatA*_ C-terminus		tgaccatgattacgccaagcctgaagcccggcgaatctc
		gtcgacctgcaggcatgcaattgatgtgggtttacttcgatcaattg
pT25		gtacctaagtaactaagaattcggc
		ccggg gatcctctagagtc
pT25_*tapA1*		cgactctagaggatccccggagtgatgaacaggagctac
		attcttagttacttaggtacgcggaacctctggaattg
pT25_*tapA2*		cgactctagaggatccccggggattgaacatagatcttgatcgtg
		attcttagttacttaggtaccatattctgtgggccggag
pT25_*eatA*		cgactctagaggatccccggccgacgaaatcagaaattc
		attcttagttacttaggtacttgatgtgggtttacttc
pT25_*eatA*_N-terminus		cgactctagaggatccccggccgacgaaatcagaaattcttaattg
		attcttagttacttaggtactcgatggccactgaagtc
pT25_*eatA*_C-terminus		cgactctagaggatccccggctgaagcccggcgaatctc
		attcttagttacttaggtacttgatgtgggtttacttcgatcaattg

Supplementary Table 3. Predicted T7 Toxin sub-types identified

Toxin C-terminal module classification	Subgroup	C-terminal module prediction	Number of sequences within dataset (%)	Annotation/description	Example Accession
A	-	DUF4185/GH183	22 (1.5)	DUF4185 domain-containing protein; PF13810, β_Fructosidase Clan; glycoside hydrolase family 183 displays endo-α-1,5-D-arabinofuranase activity[1].	WP_074339443.1
B	B1	TNT	10 (0.7)	TNT domain-containing protein; PF14021, ADP-ribosyl Clan; acts as a NAD^+ glycohydrolase triggering cell death. Putative binding pocket of Q822, Y765 and R757 has been identified in the CpnT of *M. tuberculosis*[2].	WP_228843980.1
	B2		7 (0.5)		
	B3		35 (2.4)		
C	C1	a/b hydrolase 8 family	20 (1.4)	α/β hydrolase; PF06259, α/β_hydrolase Clan; contains a predicted Ser-His-Asp catalytic triad. Family named after characteristic 8 β-sheets, family covers a diverse range of enzyme classes[3].	WP_100480021.1
	C2		48 (3.3)		
	C3		49 (3.4)		
	C4		64 (4.4)		
D	D1	NlpC/P60	84 (5.8)	NlpC/P60 family protein; PF00877, Peptidase_CA Clan; demonstrated to be involved in turnover of PG and essential to cell division[4].	WP_079641497.1
	D2		22 (1.5)		
E	-	Tox-REase-5	30 (2.1)	Tox-REase-5 domain-containing protein; PF15648, PDDEXK Clan; polymorphic toxin associated with multiple secretion systems and bacterial groups, possesses a restriction endonuclease fold (D-[EQ]xK)[5].	WP_079616854.1
F	F1	Tox-REase-7	9 (0.6)		WP_100474587.1

	F2		17 (1.2)	Putative toxin; PF15649, PDDEXK Clan; polymorphic toxin associated with multiple secretion systems and bacterial groups, possesses a restriction endonuclease fold (D-[EQ]xK)[5].	
G	G1	Ntox15	15 (1.0)	Hypothetical protein; PF15604; polymorphic toxin associated with multiple secretion systems and predicted to display RNase activity[5].	WP_235680611.1
	G2		2 (0.1)		
H	-	Ntox37	35 (2.4)	Polymorphic toxin type 37; PF15535; polymorphic toxin associated with multiple secretion systems, possesses an all-b fold and predicted to display RNase activity[5].	WP_005076669.1
J	-	Ntox44	3 (0.2)	Polymorphic toxin type 44; PF15607; polymorphic toxin associated with multiple secretion systems, possesses an all-a-helical fold and predicted to display RNase activity[5].	WP_237112254.1
K	K1	Nuclease	5 (0.3)	Colicin-DNase; PF12639, His-Me_finger Clan; endonuclease domain that targets the ribosome to inactivate protein synthesis[6].	WP_057138139.1
	K2		30 (2.1)	DNA/RNA non-specific endonuclease; PF13930, His-Me_finger Clan; possesses His-Me_finger fold observed in the *S. aureus* EsaD T7SSb antibacterial nuclease[7].	WP_005089709.1
	K3		7 (0.5)	HNH/ENDO VII superfamily / GH-E family nuclease; PF14410, His-Me_finger Clan; NucA-like nuclease associated with multiple polymorphic toxins[8].	WP_079617781.1
	K4		53 (3.7)	HNH/ENDO VII superfamily / GH-E family nuclease; PF14410, His-Me_finger Clan; NucA-like nuclease associated with multiple polymorphic toxins[8].	WP_131832996.1

L	-	Ribose hydrolase	21 (1.5)	Hypothetical protein; PF02267, Nribosyltransf Clan; domain associated with the synthesis of cyclic ADP from NAD[9].	WP_100484957.1
M	-	CdiA_C_tRNase	2 (0.1)	Hypothetical protein; PF18664, PDDEXK Clan; possesses tRNase α/β-folds associated with PD(D/E)xK nucleases. Homologous to the type II CdiA from *Burkholderia*[10].	WP_052572227.1
N	-	FGE-sulfatase	15 (1.0)	Hypothetical protein; PF03781, C_Lectin Clan; eukaryotic-associated domain involved in targeting and modification of sulfate esters, has been shown to act as an iron(II)-dependent oxidoreductase in mycobacteria[11].	WP_074330702.1
O	-	ADP-ribosyltransferase	3 (0.2)	ADP-ribosyltransferase; PF01129, ADP-ribosyl Clan; domain involved in post-transcriptional modification of proteins through transfer of NAD, homologous toxicity mechanism observed in the Cholera and Pertussis toxins[12].	WP_005095732.1
P	-	Colicin D	13 (0.9)	Colicin D; PF11429, Colicin_D_E5 Clan; displays tRNase activity through targeting the phosphodiester bonds between the stem and loop[13].	WP_016343585.1
Q	-	Amidase 6	37 (2.6)	Amidase domain-containing protein; PF12671, Peptidase_CA Clan; putative amidase domain that may act in a hydrolytic mechanism[14].	WP_052537087.1
R	-	pApp synthetase	41 (2.8)	ATP 3' pyrophosphokinase; cd05399, RelA/SpoT-like nucleotidyltransferase domain; domain associated with alarmone synthetases involved in gene regulation and co-ordinated stress responses[15].	WP_074249843.1

Note—Table Supplementary Table 3 details 699 toxin proteins comprising 48.6% of the 1,439-protein dataset. 677 (47%) proteins were classified EatU (EatU1 – U33) because their C-termini could not be assigned a predictive function. The remaining 63 (4.4%) protein sequences were removed throughout the analysis pipeline due to NCBI errors or FlaGs' rejection of accessions.

Supplementary References

1. Al-Jourani, O. *et al.* Identification of d-arabinan-degrading enzymes in mycobacteria. *Nat. Commun.* **14**, 2233 (2023).

2. Sun, J. *et al.* The Tuberculosis Necrotizing Toxin kills macrophages by hydrolyzing NAD. *Nat Struct Mol Biol* **22**, 672–678 (2015).

3. Nardini, M. & Dijkstra, B. W. α/β Hydrolase fold enzymes: the family keeps growing. *Curr. Opin. Struct. Biol.* **9**, 732–737 (1999).

4. Griffin, M. E., Klupt, S., Espinosa, J. & Hang, H. C. Peptidoglycan NlpC/P60 peptidases in bacterial physiology and host interactions. *Cell Chem. Biol.* **30**, 436–456 (2023).

5. Zhang, D., Souza, R. F. de, Anantharaman, V., Iyer, L. M. & Aravind, L. Polymorphic toxin systems: Comprehensive characterization of trafficking modes, processing, mechanisms of action, immunity and ecology using comparative genomics. *Biol Direct* **7**, 18–18 (2012).

6. Soelaiman, S., Jakes, K., Wu, N., Li, C. & Shoham, M. Crystal Structure of Colicin E3 Implications for Cell Entry and Ribosome Inactivation. *Mol. Cell* **8**, 1053–1062 (2001).

7. Cao, Z., Casabona, M. G., Kneuper, H., Chalmers, J. D. & Palmer, T. The type VII secretion system of Staphylococcus aureus secretes a nuclease toxin that targets competitor bacteria. *Nat Microbiol* **2**, 16183 (2016).

8. Zhang, D., Iyer, L. M. & Aravind, L. A novel immunity system for bacterial nucleic acid degrading toxins and its recruitment in various eukaryotic and DNA viral systems. *Nucleic Acids Res.* **39**, 4532–4552 (2011).

9. Prasad, G. S. *et al.* Crystal structure of Aplysia ADP ribosyl cyclase, a homologue of the bifunctional ectozyme CD38. *Nat. Struct. Biol.* **3**, 957–964 (1996).

10. Johnson, P. M. *et al.* Unraveling the essential role of CysK in CDI toxin activation. *Proc. Natl. Acad. Sci.* **113**, 9792–9797 (2016).

11. Seebeck, F. P. In Vitro Reconstitution of Mycobacterial Ergothioneine Biosynthesis. *J. Am. Chem. Soc.* **132**, 6632–6633 (2010).

12. Okazaki, I. J., Kim, H.-J. & Moss, J. Cloning and Characterization of a Novel Membrane-associated Lymphocyte NAD:Arginine ADP-ribosyltransferase*. *J. Biol. Chem.* **271**, 22052–22057 (1996).

13. Yajima, S. *et al.* Relation between tRNase activity and the structure of colicin D according to X-ray crystallography. *Biochem. Biophys. Res. Commun.* **322**, 966–973 (2004).

14. Dong, C. *et al.* Structural insights into the inhibition of type VI effector Tae3 by its immunity protein Tai3. *Biochem. J.* **454**, 59–68 (2013).

15. Chakraburtty, R., White, J., Takano, E. & Bibb, M. Cloning, characterization and disruption of a (p)ppGpp synthetase gene (relA) of Streptomyces coelicolor A3(2). *Mol. Microbiol.* **19**, 357–368 (1996).
