# Supplementary material for: A Type VII-secreted toxin enables inter-mycobacterial competition
Source: Nat Commun. 2026 Jun 23;17:7899. doi: 10.1038/s41467-026-74429-7 (PMC13444075; doi:10.1038/s41467-026-74429-7)
Supplement: Supplementary file 2 [file 41467_2026_74429_MOESM4_ESM.pdf]

Description of Additional Supplementary Files:

Supplementary Data 1. AlphaFold models.

Supplementary Software 1. R script used for fluorescence data analysis.
