# Supplementary material for: A Type VII-secreted toxin enables inter-mycobacterial competition
Source: Nat Commun. 2026 Jun 23;17:7899. doi: 10.1038/s41467-026-74429-7 (PMC13444075; doi:10.1038/s41467-026-74429-7)
Supplement: Supplementary file 3 [file 41467_2026_74429_MOESM5_ESM.pdf]

nature portfolio

Corresponding author(s): Patrick Moynihan, Tracy Palmer

Last updated by author(s): May 25, 2026

Reporting Summary

Nature Portfolio wishes to improve the reproducibility of the work that we publish. This form provides structure for consistency and transparency in reporting. For further information on Nature Portfolio policies, see our Editorial Policies and the Editorial Policy Checklist.

Statistics

For all statistical analyses, confirm that the following items are present in the figure legend, table legend, main text, or Methods section.

n/a	Confirmed	
☐	☒	The exact sample size (*n*) for each experimental group/condition, given as a discrete number and unit of measurement
☐	☒	A statement on whether measurements were taken from distinct samples or whether the same sample was measured repeatedly
☐	☒	The statistical test(s) used AND whether they are one- or two-sided<br>*Only common tests should be described solely by name; describe more complex techniques in the Methods section.*
☒	☐	A description of all covariates tested
☐	☒	A description of any assumptions or corrections, such as tests of normality and adjustment for multiple comparisons
☒	☐	A full description of the statistical parameters including central tendency (e.g. means) or other basic estimates (e.g. regression coefficient) AND variation (e.g. standard deviation) or associated estimates of uncertainty (e.g. confidence intervals)
☒	☐	For null hypothesis testing, the test statistic (e.g. *F*, *t*, *r*) with confidence intervals, effect sizes, degrees of freedom and *P* value noted<br>*Give P values as exact values whenever suitable.*
☒	☐	For Bayesian analysis, information on the choice of priors and Markov chain Monte Carlo settings
☒	☐	For hierarchical and complex designs, identification of the appropriate level for tests and full reporting of outcomes
☒	☐	Estimates of effect sizes (e.g. Cohen's *d*, Pearson's *r*), indicating how they were calculated

Our web collection on statistics for biologists contains articles on many of the points above.

Software and code

Policy information about availability of computer code

Data collection	Alphafold 3, accessed at https://alphafoldserver.com<br>Structural data were collected at Diamond Light source using the iISPyB server<br>Unicorn 7.2<br>GraphPad Prism 10.4.1<br>Chromeleon™ Chromatography Management System V.6.8<br>EOS Utility (Canon, version 2.10.0.0)<br>S&P Imager (S&P Robotics, version 2.0.1.0)
Data analysis	ChimeraX 1.9 was used for figure generation and analysis of structural data<br>Alphafold predictions were analysed using FoldTree (google collab: https://colab.research.google.com/github/DessimozLab/fold_tree/blob/main/notebooks/Foldtree.ipyn)<br>Genetic data analysed in Geneious Prime 2024.0.7<br>Genetic data analysed in blastn (v2.13.0) and hmmer (v3.3.2)<br>Pinning analysis was completed using the following R packages: imager, magick, ggplot2, reshape2, and ggbeeswarm<br>Protein sequences analysed with SignalP 6.059<br>Genetic data analysed using DeepTMHMM (v1.0.24)<br>Genetic data analysed in MEGA (v10.1.8)

Structural data were analysed using CCP4i v1.1.0, coot v.0.9.8.1 and Phenix 1.21.2-5419-000.

For manuscripts utilizing custom algorithms or software that are central to the research but not yet described in published literature, software must be made available to editors and reviewers. We strongly encourage code deposition in a community repository (e.g. GitHub). See the Nature Portfolio guidelines for submitting code & software for further information.

Data

Policy information about availability of data

All manuscripts must include a data availability statement. This statement should provide the following information, where applicable:

- Accession codes, unique identifiers, or web links for publicly available datasets
- A description of any restrictions on data availability
- For clinical datasets or third party data, please ensure that the statement adheres to our policy

Atomic coordinates were deposited in the PDB with accessioncodes 9MXM, 9MYT and 9MZ5. The genome sequence of M. abcsessus ΔeccC4 is deposited to the ENA as project PRJEB85598.

Research involving human participants, their data, or biological material

Policy information about studies with human participants or human data. See also policy information about sex, gender (identity/presentation), and sexual orientation and race, ethnicity and racism.

Reporting on sex and gender	N/A for experiments used
Reporting on race, ethnicity, or other socially relevant groupings	N/A for experiments used
Population characteristics	N/A for experiments used
Recruitment	N/A for experiments used
Ethics oversight	N/A for experiments used

Note that full information on the approval of the study protocol must also be provided in the manuscript.

Field-specific reporting

Please select the one below that is the best fit for your research. If you are not sure, read the appropriate sections before making your selection.

☒ Life sciences ☐ Behavioural & social sciences ☐ Ecological, evolutionary & environmental sciences

For a reference copy of the document with all sections, see nature.com/documents/nr-reporting-summary-flat.pdf

Life sciences study design

All studies must disclose on these points even when the disclosure is negative.

Sample size	No calculation was performed to predetermine sample size. Sample sizes were chosen based on methods that have produced reliable and statistically-robust data previously. For pinning experiments the sample size is determined partially by plate geometry and the number of strains being observed on a given plate, always keeping n>90.
Data exclusions	No data was excluded from analysis
Replication	For TLC analysis two biological replicates were used. For all other data, at least three independent biological repeats were carried out, consistently reproducing findings. In all cases for pinning assays the number of biological replicates vastly exceeds that which is normally done for colony assays in mycobacteria (typically n=3).
Randomization	Colonies or plaques of bacteria were selected at random from plates for culture for experiments, grown separately and on different days for each biological repeat. Controlling for covariates is not applicable to the experiments carried out.
Blinding	Since no group allocation was performed in our study, blinding was not relevant.

Reporting for specific materials, systems and methods

We require information from authors about some types of materials, experimental systems and methods used in many studies. Here, indicate whether each material, system or method listed is relevant to your study. If you are not sure if a list item applies to your research, read the appropriate section before selecting a response.

Materials & experimental systems

n/a	Involved in the study	
☐	☒	Antibodies
☒	☐	Eukaryotic cell lines
☒	☐	Palaeontology and archaeology
☒	☐	Animals and other organisms
☒	☐	Clinical data
☒	☐	Dual use research of concern
☒	☐	Plants

Methods

n/a	Involved in the study	
☒	☐	ChIP-seq
☒	☐	Flow cytometry
☒	☐	MRI-based neuroimaging

Antibodies

Antibodies used

mouse-α-His (GenScript; Cat.No. A00186)
custom α-EsxU antibodies (Davids Biotechnologie)
Goat anti-mouse IgG HRP conjugate (BioRad: 1706516)
goat anti-rabbit IgG HRP conjugate (Biorad: 1706515)
α-HiBit antibody (Promega: N7200)

Validation

mouse-α-His - Publciations reported on manufacturer's website: https://www.genscript.com/antibody/A00186-THE_His_Tag_Antibody_mAb_Mouse.html
α-EsxU - Antibodies were tested against purified protein samples to confirm activity and binding specificity

Plants

Seed stocks

N/A for experiments used (this part of the form is not working correctly, no plants used)

Novel plant genotypes

N/A for experiments used (this part of the form is not working correctly, no plants used)

Authentication

N/A for experiments used (this part of the form is not working correctly, no plants used)
